# Sources of gut microbiota variation in a large longitudinal Finnish infant cohort

**DOI:** 10.1016/j.ebiom.2023.104695

**Published:** 2023-07-01

**Authors:** Roosa Jokela, Alise J. Ponsero, Evgenia Dikareva, Xiaodong Wei, Kaija-Leena Kolho, Katri Korpela, Willem M. de Vos, Anne Salonen

**Affiliations:** a Human Microbiome Research Program, Faculty of Medicine, University of Helsinki, Helsinki, Finland; b Children's Hospital, Paediatric Research Centre, University of Helsinki and HUS, Helsinki, Finland; c Faculty of Medicine and Health Technology, Tampere University, Tampere, Finland; d Laboratory of Microbiology, Wageningen University, Wageningen, the Netherlands

**Keywords:** Infant gut microbiota, Birth cohort, Variance partitioning, Parity, Stool consistency, Technical confounders

## Abstract

**Background:**

Although the infant gut microbiota has been extensively studied, comprehensive assessment on the microbiota determinants including technical variables has not been performed in large infant cohorts.

**Methods:**

We studied the effect of 109 variables on the 16S rRNA gene amplicon-based gut microbiota profiles of infants sampled longitudinally from three weeks to two years of life in the Finnish HELMi birth cohort. Spot faecal samples from both parents were included for intra-family analyses, totalling to 7657 samples from 985 families that were evaluated for beta-diversity patterns using permutational multivariate analysis on Bray–Curtis distances, and differential abundance testing and alpha-diversity for variables of interest. We also assessed the effect of different taxonomic levels and distance methods.

**Findings:**

In time point-specific models, the largest share of variation explained, up to 2–6%, were seen in decreasing order for the DNA extraction batch, delivery mode and related perinatal exposures, defecation frequency and parity/siblings. Variables describing the infant gastrointestinal function were continuously important during the first two years, reflecting changes in *e.g.*, feeding habits. The effect of parity/siblings on infant microbiota was modified by birth mode and exposure to intrapartum antibiotics, exemplifying the tight interlinkage of perinatal factors relevant for infant microbiota research. In total, up to 19% of the biological microbiota variation in the infant gut could be explained. Our results highlight the need to interpret variance partitioning results in the context of each cohort's characteristics and microbiota processing.

**Interpretation:**

Our study provides a comprehensive report of key factors associated with infant gut microbiota composition across the two first years of life in a homogenous cohort. The study highlights possible important future research areas and confounding factors to be considered.

**Funding:**

This research was supported by 10.13039/501100014438Business Finland, 10.13039/501100002341Academy of Finland, Foundation for Nutrition Research and the Doctoral Program in Microbiology and Biotechnology, University of Helsinki, Finland.

**Research in context**

**Evidence before this study**

Infant microbiota development is under intensive research due to its importance for early immunological and other child development. Current infant microbiota research has focused on studying the effect of individual variables, such as birth mode and breastfeeding, or characterising the microbiota of infants with specific health issues such as allergy. Comprehensive assessments of how technical factors, early exposures and health variables associate to infant gut microbiota variation in a large-scale cohort has been lacking.

**Added value of this study**

Utilising the 6378 longitudinal microbiota samples and 109 covariates collected from 985 infants in the Finnish HELMi birth cohort, we asked to which extent each of the variables explains infant microbiota variation using Bray-Curtis dissimilarity as a measure of beta-diversity. Altogether up to 19 to 50% of the variance could be explained depending on whether the eight sampling points were analysed separately or as pooled. The main effectors varied according to infant age, but overall, the key covariates were perinatal exposures, stool frequency and form, family size and the sample processing batch. The selected statistical methods, and variables used did not allow for precision to see associations between child health, medication or probiotic use on the overall microbiota variance in this homogenous cohort.

**Implications of all the available evidence**

Variance partitioning on large cohorts provides a powerful tool to identify microbiota covariates and their effect sizes for further, targeted research *e.g.*, as a potential microbiota modulator or a confounding factor. The cohort characteristics and homogeneity affect the results and need to be taken into account when interpreting and comparing the results to those of the other cohorts. Our results emphasise the need to account for technical factors also in single-centre large human microbiome studies. Many of the perinatal factors were associated with the delivery mode, implying that mother and infant microbiota studies should preferentially use stratification over model adjustment for the birth mode not to unintentionally remove the effect of perinatal factors of potential interest.

## Introduction

Infant gut microbiota colonisation and development, *i.e.*, the gradual transition from a neonatal phase to a more adult like composition during the first years of life,^1^^,^^2^ are influenced by a complex interplay between the host and external factors. Neonatal colonisation starts at birth^3^ and is greatly affected by delivery mode^4^, ^5^, ^6^, ^7^ and early antibiotic exposures.^8^^,^^9^ In industrialised populations, neonate's first microbial exposures usually happen in the hospital. The impact of these perinatal factors is known to affect the infant gut microbiota composition for months after birth.^10^ Additionally, infant diet, especially exclusive breastfeeding^11^ but also the timing of introduction of solid foods^12^ and the cessation of breastfeeding,^13^ affect infant gut microbiota maturation. Maternal factors will affect the microbes transferred to the infant at delivery and later in infancy through breastfeeding and other interactions. Hence, several maternal factors both pre- and postnatal, such as diabetes,^14^ body mass index (BMI),^14^^,^^15^ and diet,^14^^,^^16^ have been associated with infant gut microbiota composition and development. Comprehensive understanding of the relative impact of factors that affect infant gut microbiota composition may contribute to developing practices that support a beneficial gut microbiota development.

While most research has focused on understanding the individual impact of a single factors that affect the infant gut microbiota development, few studies compared the effects of different peri- and postnatal exposures and factors on the microbiota composition at different ages.^1^^,^^17^^,^^18^ In the largest study so far, Stewart *et al.*^18^ reported breastfeeding as the most important variable explaining infant gut microbiota composition during the first year of life out of 22 variables tested in a cohort of 903 infants from Europe and USA. This study also identified geographical location, presence of siblings or furry pets in the household, birth mode and infant probiotic intake to significantly impact the microbiota variation at several ages during the first two years of life. Strikingly, each individual variable explained at most approximately 1% of the species level compositional variation. Wernroth *et al.*^2^ confirmed the transient effect of delivery mode on neonates’ gut microbiota, but saw no other associations with the variables tested and the infant gut microbiota composition except birth weight at the age of 1 year. These studies suggested a complex and dynamic impact of the different effectors on the infant gut microbiota during infancy that requires large scale longitudinal cohorts to be fully explored.

Notably, large cohort or population wide microbiota studies rarely report the impact of technical variation and compare it to the biological factors of interest, although the impact and importance of technical variation in human gut microbiota studies is well known *e.g.*, for sample storage,^19^ DNA extraction,^20^ and library preparation.^21^ In larger sample sets and longer studies, despite adapting standard operating procedures there is an inherent variation in the lab personnel and reagent lots, and the samples are typically extracted and sequenced in batches. However, the relative importance of these factors has not been addressed systematically. In addition, there is limited transparency in reporting the magnitude of the effect from different technical variables apart from methodical papers. However, this is highly important as exemplified by a recent study where sequencing run batch accounted for up to 8% of the total microbial variation in Kenyan infants aged 0–9 months.^22^ Understanding the role of technical variation will help to identify potential misclassification and bias and separate technical from biological effects on the microbiota composition.

In this study, we explore an extensive set of both technical and biological effectors and their relative importance on the different stages of infant gut microbiota development. We leverage the Health and Early Life Microbiota (HELMi) birth cohort,^23^ which includes the 16S rRNA gene amplicon sequenced faecal samples from 985 infants from the first 2 years of life and samples from both parents, and 109 variables collected from questionnaires and hospital records detailing the perinatal conditions.

## Methods

### Sample and data collection

The HELMi cohort (NCT03996304)^23^ comprises 1055 families recruited from the general population mainly from the metropolitan region of Finland during 2016–2018. Healthy, term, singleton babies born on gestational weeks 37–42 with birth weight exceeding 2.5 kg were included in the cohort. At least one of the parents had to speak Finnish to be able to answer the extensive electronic questionnaires. Eight stool samples collected from the infants for microbiota characterisation during the first two years and spot samples from the parents collected near the delivery were used in this study. The stool samples were collected at 3, 6 and 12 weeks, and 6, 9, 12, 18, and 24 months and stored at home at −20 °C, transported to the laboratory in frozen form and kept at −80 °C until DNA extraction.

Data on nutrition, growth, health and well-being, environment and lifestyle, and maternal and parental factors were collected with online questionnaires at different time intervals from weekly to one-time questionnaires, from a prenatal background questionnaire to a questionnaire after the child's 2-year birthday.^23^ The mode of delivery, intrapartum antibiotics, maternal group B streptococcus (GBS) status, and the timing of rupture of membranes were collected from the hospital records.^24^ Delivery mode and the use of intrapartum antibiotics were combined to a three-class delivery variable (Caesarean section (C-section) with intrapartum antibiotics, vaginal delivery with or without intrapartum antibiotics) to test also their combined effect. The list of variables, methods of aggregation, and exclusion criteria are presented in Supplemental File S1. Technical variables used were collected at the laboratory, *e.g.*, DNA yield was calculated based on the faecal mass used for extraction and the DNA concentration attained.

Families with infants with a diagnosis of a serious long-term illness (other than allergic diseases) by 2 years were excluded (N = 31). Additionally, in families with two female parents, the non-biological mother was excluded (N = 8) from this study. This study had both biological and non-biological fathers (N = 6), both referred as fathers from here on. Variables describing the socioeconomic background (*e.g.*, level of education) addressed the acting guardian, whereas variables on health and disease history were linked to the child's biological father (more precisely itemised in Supplemental File S1). Categorical variables with extremely imbalanced group sizes (all but one category with a group size of <10 by sample type), and variables with a large proportion of missing values (>75% of the samples with missing values at a specific time point) were excluded from the analysis.

### Ethics statement

The cohort study was approved by The Hospital District of Helsinki and Uusimaa (263/13/03/03 2015) and performed in accordance with the principles of the Helsinki Declaration. Parents signed an informed consent at enrolment.

### DNA extraction and 16S rRNA gene amplicon sequencing

DNA was extracted from the stool samples using a bead-beating method.^20^ In short, *ca.* 250 or 340 mg of faecal material was suspended in 0.5 or 1 ml of sterile ice-cold PBS, and 250 μl of faecal suspension was combined with 340 μl of RBB lysis buffer (500 mM NaCl, 50 mM Tris-HCl (pH 8.0), 50 mM EDTA, 4% SDS) in a bead-beating tube from the Ambion MagMAX™ Total Nucleic Acid Isolation Kit (Life Technologies). After repeated bead-beating, 200 μl of the supernatant was used for DNA extraction with a KingFisherTM Flex automated purification system (ThermoFisher Scientific) using a MagMAXTM Pathogen High Vol. DNA was quantified using Quanti-iT™ Pico Green dsDNA Assay (Invitrogen). The V3–V4 region primers (primers 341F/785R) of the 16S rRNA gene was amplification was performed according to the Illumina manual with slight modification for TrusSeq-tailed 1-step amplification.^25^ For infant samples until 12 months, 5 ng of DNA template was used for index-PCR (27–40 cycles) and 1 ng from thereafter and for adult samples. The library preparation protocol has been described in more detail previously.^26^ Sequencing was performed with Illumina MiSeq and HiSeq sequencing technology in the Functional Genomics Unit and Institute for Molecular Medicine Finland, University of Helsinki, Helsinki, Finland.

### Quality control and taxonomic profiling

The sequencing reads were processed using the R package Dada2 for quality filtering, chimera detection, and taxonomic annotation.^27^ Sequencing depth cut-off was set to 3000 processed paired reads to samples collected at 3 months or before and to 5000 paired reads for the remaining samples based on species richness to sequencing depth evaluations. The raw reads were deposited and made available with critical metadata in the European Nucleotide Archive (ENA, PRJEB55243).

Taxonomic annotation was performed using Dada2 against the RefSeq 16S rRNA database available at https://doi.org/10.5281/zenodo.2541238, using the assignTaxonomy function which implements a naive Bayesian classifier method provided by the Ribosomal Database Project.^28^ A minimum bootstrapping support threshold of 50 was used. Annotations at genus level were used for the analyses and unknown annotations were removed from the data. Finally, samples with over 20% of unknown annotations were excluded from the analysis.

### PERMANOVA modelling

The permutational analysis of variance (PERMANOVA) was performed in R using the vegan package function adonis2^29^ by sampling age. Bray–Curtis distance was calculated between genus-level-aggregated relative abundances for each sample. Bray–Curtis is a compositional distance method that accounts for both presence and abundance of taxa in the communities (BC = 1−(2C_ij_/(S_i_ + S_j_)), where C_ij_ is the sum of the lowest abundance of each taxon in the two communities and S_i_ and S_j_ are the total number of taxa present in the two communities). *P*-values under 0.05 were considered statistically significant if the false discovery rate (FDR) value was under 0.1. Based on the analysis of the variation explained by the technical variables, DNA extraction plate number was chosen as a confounder to all subsequent analysis. Additionally, the number of processed reads was used as a second confounder in PERMANOVAs, to account for the sampling effect that stem from variations in the sequencing depth. DNA extraction plate was handled as a categorical variable and the number of processed reads as a continuous variable. In this study, only technical variables were considered confounders, possibly affecting the perceived variation explained by the other variables in question. All tested variables were plotted but are distributed between the main text and supplemental material based on their relevance.

The cumulative model parameters were selected based on the per time point individual PERMANOVA results and biologically relevant variables, whereafter backward selection using Akaike information criterion (AIC) for small sample sizes due to the large number of parameters (2 × k + n + ln (RSS/n) + (2 × k × (k + 1))/(n − k − 1), where k = number or parameters, n = number of samples, RSS = residual sum of squares) was conducted, confounding with the DNA extraction plate and number of processed reads. The models also included interactions between two variables (var 1:var 2), that were found statistically significant in a separate PERMANOVA, detailed in Supplemental File S2. To simplify the models, the three-level delivery mode was used in the cumulative models. A model was deemed better if the new AIC was at least two units lower than the previous one. Another PERMANOVA model with all infant samples from all ages, with only the number of processed reads as a confounder, was constructed to identify the key explanatory variables across all time points. Similar models were constructed including the technical variables by time point and parental samples to assess the total effect of technical variables, but no model selection was conducted to identify the maximum amount of technical variation in this cohort.

### Other statistical methods

Alpha-diversity measure (Shannon index), richness (Chao 1 index), community evenness (Pielou index), and rarity (log modulo skewness index) were estimated with the R vegan^30^ and microbiome packages,^31^ on the taxonomic profiles aggregated at the genus level after rarefying to the smallest number of processed reads. Comparison between groups were performed using unpaired Wilcoxon test and *P*-values <0.05 were considered as statistically significant. When a pairwise Wilcoxon test was carried out on more than two groups of samples, an FDR correction was applied, and FDR adjusted *P*-values <0.05 were considered as statistically significant.

The differential abundance testing of taxa was conducted with the R package mare,^32^ utilising the functions from MASS,^33^ nlme,^34^ and glmmADMB^35^ packages. The analyses were conducted primarily by negative binomial models, or by Poisson, quasi-Poisson or generalised least squares models, depending on data distribution and model fit using the number of processed reads as an offset. The models were adjusted for the DNA extraction batch, except for DNA yield that was adjusted with stool consistency and sequencing platform where no adjustment was done. Only genera present in >10% of the samples were analysed individually (22–77 individual genera), only *P*-values <0.05 with an FDR <0.1 were considered significant and only significant results were presented in the text. For categorical values, the reference category, the group against which other groups were compared, was selected either based on what was considered more standard (*e.g.*, vaginal delivery *versus* C-section delivery) or the lowest category (*e.g.*, defecation rates).

Correlations between numerical variables were assessed using Spearman rank correlation from the stats R package, and correlation with a coefficient (R^2^) above 0.3 or below −0.3 were considered fair.^36^

Normalised mutual information (symmetric uncertainty) was computed to assess possible dependencies between numeric and categorical variables using the infotheo v1.2.0 R package^37^ and was computed as: (2 × Mutual information (Var1, Var2))/(Entropy (Var1) + Entropy (Var2)).

### Role of funders

The funders were not involved in study design, data collection, analysis, interpretation, or writing.

## Results

### Cohort overview

Faecal samples from the HELMi cohort^23^ were obtained and processed for sequencing from 1019 infants. The current study comprises samples from 985 infants with gut microbiota sequences meeting the quality criteria (see Methods), 824 born via vaginal delivery and 161 via C-section. All C-section and 192 vaginal deliveries involved intrapartum antibiotics. An overview of the background and early exposure characteristics of the selected cohort samples stratified by birth mode is presented in Supplemental Table S1. The cohort was mostly composed of highly educated families (88% of mothers with a higher education) and 49% of the infants were first-born.

Infant stools were sampled at 3, and 6 weeks, and 3, 6, 9, 12, 18, and 24 months of age, with an average of 6 samples per infant (Supplemental Table S1). We also included parental samples from 830 families, 720 from mothers and 559 from fathers, collected around the delivery date (median time: 2 days before delivery). In total, 6378 infant samples and 1279 parental samples were included.

**Fig. 1:**
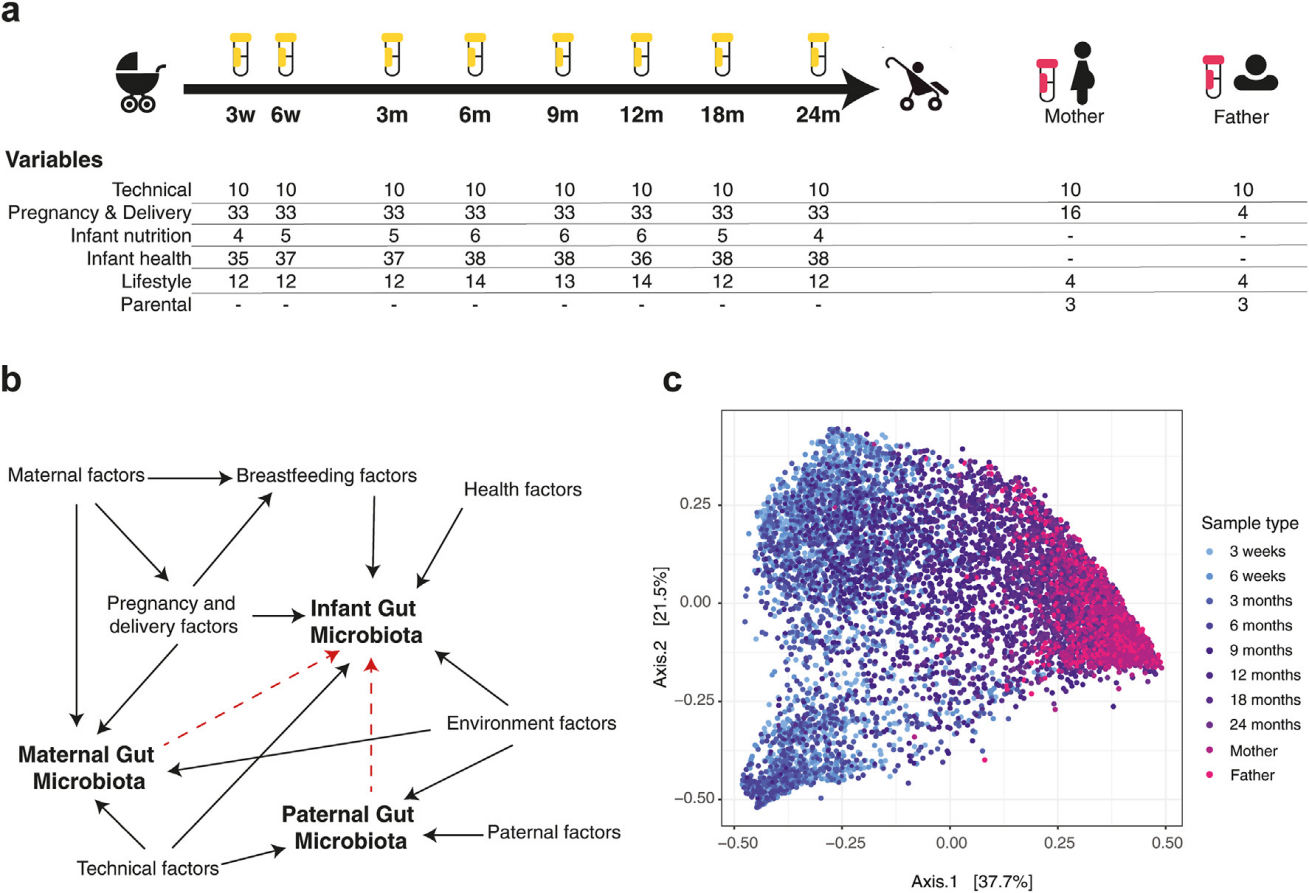
**Study overview.** (a) Overview of the samples and number of variables per metadata category and sampling age and the parental samples, (b) directed acyclic graph of the interactions between maternal (*e.g*., parity, diabetes), paternal (*e.g.*, allergy, body mass index), breastfeeding, health (*e.g.*, antibiotics, gastrointestinal function), and environmental (*e.g.*, pets, cleaning) and technical (*e.g.*, sequencing run, storage) variables and gut microbiota composition, red dotted arrows representing potential microbiota transmission patterns inferred from previous literature, and (c) principal coordinate analysis on Bray–Curtis distances of all the samples. w, weeks, m, month. A detailed description of the variables, their definitions and groupings are available in Supplemental File S1.

In total 109 variables regarding pregnancy and birth conditions, lifestyle, infant diet, and health (Fig. 1a) were used to characterise the cohort (Supplemental File S1). Fig. 1b graphically depicts the investigated variable categories and their assumed reciprocal associations and their associations with the outcome of interest: the infant microbiota. Association between birth mode and other variables are presented in supplemental results and Supplemental Fig. S1. After sequencing, a median of 16.8k processed high-quality reads were obtained per sample (median 16.6k reads in infant and 17.5k in parental samples). As expected, a global convergence of infant microbiota toward an adult-like composition (Fig. 1c, Supplemental Fig. S2a), as well as an increase in taxonomic alpha-diversity was observed during the two first years of life (Supplemental Fig. S2b).

### Role of technical variables in faecal microbiota variance in infants and their parents

**Fig. 2:**
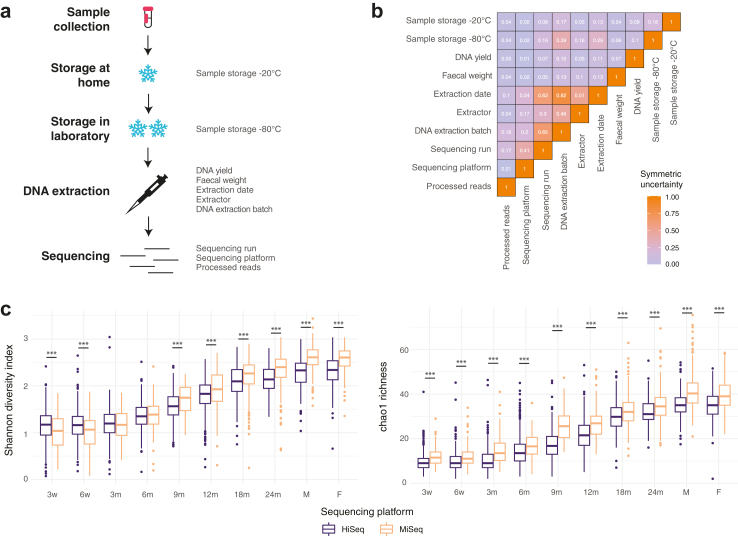
**Interactions of technical variables and effects on observed microbiota diversity and richness.** (a) Overview of technical variables, (b) normalised mutual information (symmetric information) between technical variables to evaluate the information redundancy of the variables, and (c) effect of sequencing platform on alpha-diversity and richness by sampling age and parental samples. Asterisks mark *P*-values between the two platforms. ∗∗∗: *P* < 0.001, ∗∗: *P* < 0.01, ∗: *P* < 0.05, -: *P* < 0.1. w = weeks, m = month, F = father, M = mother.

First, we assessed the variation caused by technical variables to characterise their impact and identify potential confounders for the subsequent analysis. We included 10 technical variables for infants' and parents’ samples. The variables covered the possible effects arising from sample storage, DNA extraction, and the sequencing process (Fig. 2a, Supplemental File S1). The DNA extraction was performed in 96-well format in 98 batches by 5 different extractors following a rigorously standardised beat-beating protocol. The extractions were performed over a period of 43 months, making the date of extraction, DNA extraction batch, and extractor highly interlinked variables (Fig. 2b). Associations between the different technical variables and technical variables and alpha-diversity measures as well as genus levels associations with DNA yield (Supplemental Fig. S3) are more extensively covered in the supplemental material.

The 7657 samples were sequenced in 16 runs using two sequencing platforms from Illumina; after discontinuation of the HiSeq 2500 system, sequencing was continued with MiSeq, resulting in interactions between the sequencing platform and the DNA extraction variables. The library preparation laboratory, protocols, and personnel remained the same. MiSeq resulted a lower number processed reads than HiSeq (Wilcoxon, *P* < 0.05, stratified by sample type; mean read count for adult samples: 19k for HiSeq and 16k for MiSeq) and, even after rarefying all samples at a comparable depth, the platform had a significant impact on the alpha-diversity, richness, evenness, and rarity in most sample types (Wilcoxon, *P* < 0.05, stratified by sample type) (Fig. 2c, Supplemental Fig. S4a, and b). Further, the difference in the number processed reads was reflected in differences in the relative abundance of several genera (Supplemental Fig. S4d).

**Fig. 3:**
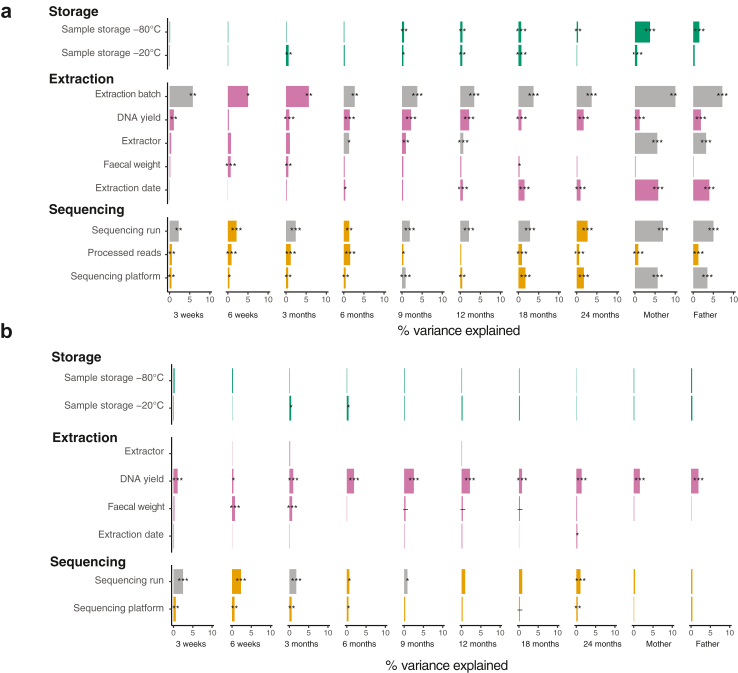
**Variation explained by technical variables and DNA extraction yield** (a) before and (b) after adjusting for DNA extraction batch and number of processed reads. Results depict the coefficient of determination (R^2^) from permutational multivariate analysis on Bray–Curtis distances. Asterisks mark false discovery rate corrected *P*-values. ∗∗∗: *P* < 0.001, ∗∗: *P* < 0.01, ∗: *P* < 0.05, -: *P* < 0.1, all non-corrected *P*-values <0.05. Grey bars mark uneven beta-dispersion between the categories of a variable. A detailed description of the variables, their definitions and groupings are available in Supplemental File S1.

Next, we assessed the variation explained by each technical variable using PERMANOVA on Bray–Curtis distances. DNA extraction batch was both highly significant and explained the largest part of the microbiota variation at all sampling times, at most 10% in mothers' samples and from 2.7% to 5.8% in infant samples (Fig. 3a). Samples from the same time point extracted in the same DNA extraction batch had a significantly higher microbiota similarity than samples extracted in different batches (Wilcoxon test, *P* < 0.05 on Bray–Curtis distance). The variation explained by technical variables was globally more important in adult samples, most notably the effect of the extractor, date of extraction, sequencing run, and sequencing platform. To assess the combined effect of technical variables on the microbiota variations, we constructed PERMANOVA models including all technical variables by sample type. The cumulative variance explained by the technical variables on the bacterial composition ranged between 6.8% and 15% in infant samples, peaking at 3 months, and explained 13% and 11% in mothers' and fathers’ samples, respectively (Supplemental Fig. S5).

To account for the impact of extraction and sequencing variables on the compositional variation, we chose the DNA extraction batch and the number of processed reads as confounders for the rest of the analyses. After this adjustment, the effect size of all technical variables decreased substantially, not exceeding 3% for any of the technical variables (Fig. 3b).

We finally evaluated the impact of different distance methods and taxonomic levels used in the analysis by repeating the PERMANOVAs of the separate technical variables using Pearson's and Aitchison distances on the genus level and Bray–Curtis distance (measure otherwise used in this study) on family level (Supplemental Fig. S6). Although the main patterns in the results were comparable, some associations and effect sizes varied between the methods. For example, effect sizes were generally smaller for genus (Supplemental Fig. S6a) than family level (Supplemental Fig. S6b) compositions, especially in the parental samples. Effect sizes were also generally slightly smaller with Pearson's (Supplemental Fig. S6c) and Aitchison (Supplemental Fig. S6d) distance. For the rest of the study, the individual and cumulative variance explained by biological variables was investigated using PERMANOVA on the widely established Bray–Curtis distances, adjusted for both DNA extraction batch and number of processed reads.

### Impact of pregnancy and delivery variables

**Fig. 4:**
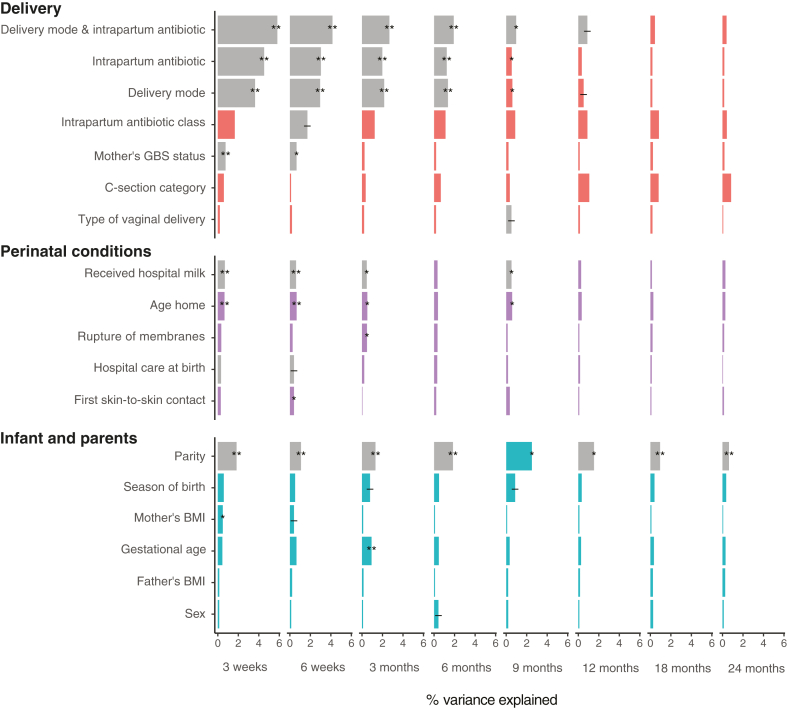
**Variance explained in the infant microbiota by birth and pregnancy variables by sampling age.** Results depict the coefficient of determination (R^2^) from permutational multivariate analysis on Bray–Curtis distances after adjustment with extraction batch and number of processed reads. Asterisks mark false discovery rate corrected *P*-values. ∗∗∗: *P* < 0.001, ∗∗: *P* < 0.01, ∗: *P* < 0.05, -: *P* < 0.1, all non-corrected *P*-values <0.05. Grey bars mark uneven beta-dispersion between the categories of a variable. A detailed description of the variables, their definitions and groupings are available in Supplemental File S1.

Next, we assessed the impact of pre- and perinatal factors on the infant microbiota composition. A total of 33 variables were investigated, covering maternal health and exposures during pregnancy, delivery mode and conditions, as well as parental characteristics adjusting for DNA extraction batch and the number of processed reads (Supplemental File S1, Fig. 4, and Supplemental Fig. S7). Delivery mode (vaginal or C-section delivery) and exposure to intrapartum antibiotics (yes/no) were more important in the early time points reducing in importance as the infant got older (Fig. 4), and the combined three-level delivery variable explained 5.8% of the variation at 3 weeks and decreased to 0.4% by 2 years.

As expected, the effects of C-section delivery and vaginal delivery with intrapartum antibiotics (compared to vaginal delivery without antibiotics) on taxon abundance were similar, but the associations were stronger in the C-section delivered infants (Supplemental Fig. S8a). For example, the relative abundance of *Bacteroides* was reduced more drastically in C-section delivered infants and it was observed still at 12 months whereas the last significant association in the antibiotic exposed vaginally delivered infants was at 6 months. Also, lower levels of *Parabacteroides* (until 6 months) and higher levels of *Salmonella* (until 9 months) were mainly detected in C-section delivered infants compared to vaginally delivered non-exposed infants. However, the impact of birth mode and intrapartum antibiotic exposure on the alpha-diversity or genus richness was not significant except at 9 months where richness was significantly increased in C-section delivered infants (Wilcoxon, *P* < 0.05, stratified by time point) (Supplemental Fig. S8b and c).

**Fig. 5:**
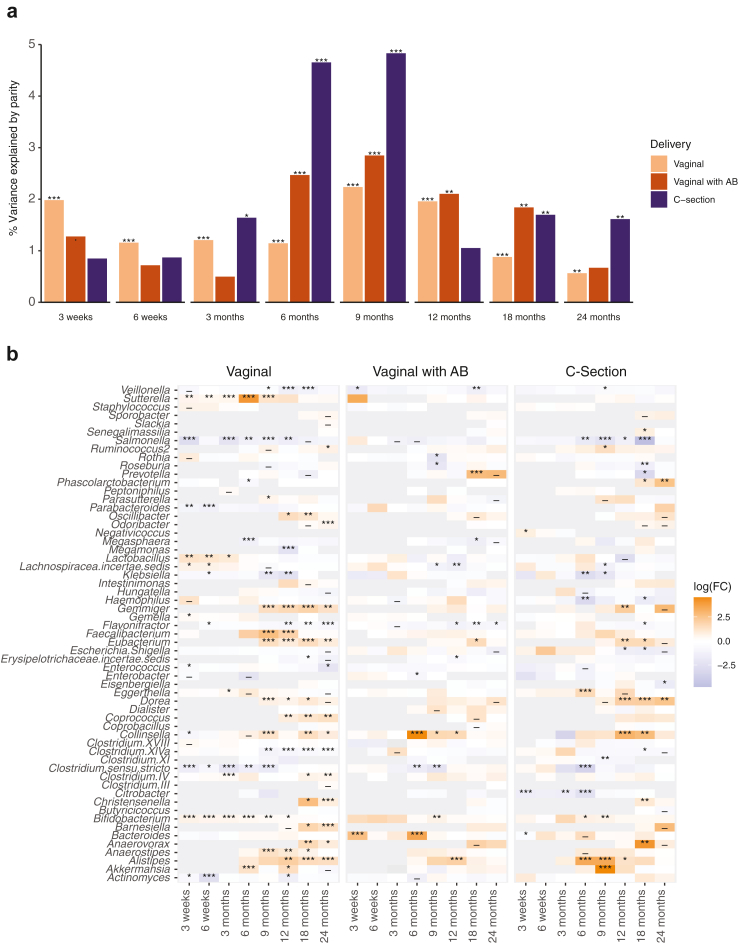
**Impact of the maternal parity stratified by the mode of delivery and exposures to intrapartum antibiotics on the (a) variance explained in the infant microbiota and (b) relative abundances of different genera by sampling age.** Results depict the coefficient of determination (R^2^) from permutational multivariate analysis on Bray–Curtis distances after adjustment with extraction batch and number of processed reads (a). Infants born to multiparous mothers were used as the reference group, meaning orange colours symbolise positive and purple negative association with nulliparity (b). Asterisks mark raw *P*-values (a) or false discovery rate corrected *P*-values (b). ∗∗∗: *P* < 0.001, ∗∗: *P* < 0.01, ∗: *P* < 0.05, -: *P* < 0.1, all non-corrected *P*-values <0.05. A detailed description of the variables, their definitions and groupings are available in Supplemental File S1. AB = antibiotic, FC = fold change.

Generally, maternal health or exposures during pregnancy and parental characteristics had a negligible effect on the overall composition of the infant gut microbiota (Fig. 4 and Supplemental Fig S7). An interesting exception was parity (analysed as binary variable; mothers with or without previous deliveries, highly correlating with presence of older siblings) that explained 0.6–2.5% of the microbiota variation in infants’ samples during the first 2 years. The analysis was also conducted with parity as numerical variable with comparable results (data not shown). The effect of parity was age-dependent: before 3 months, multiparity was significantly associated with increased alpha-diversity and community evenness, while after 12 months, a decrease in both alpha-diversity and genus richness was observed (Wilcoxon, *P* > 0.05, stratified by time point) (Supplemental Fig. S9a–d). As delivery mode and parity were significantly associated (Supplemental Fig. S1a), we performed a stratified analysis taking into account the delivery mode and intrapartum antibiotic exposure. Until 3 months, parity was significantly associated with the microbiota composition in non-antibiotic exposed vaginally delivered infants only (Fig. 5a). However, around 6–9 months, the effect of parity was highest in the C-section-delivered infants, explaining close to 5% of the variance. In genus level analysis, most differences between nulli- and multiparous mothers were seen in vaginally delivered, non-exposed infants, especially during the first 6 months (Fig. 5b). Notably, *Parabacteroides*, *Actinomyces*, *Clostridium sensu stricto*, and *Salmonella* were reduced early in the infants of nulliparous mothers, with the reduction of *Clostridium sensu stricto* and *Salmonella* lasting until 9 and 18 months, respectively. On the other hand, *Lactobacillus*, *Sutterella,* and *Bifidobacterium* were observed in higher relative abundances in the nulliparous group, and the association was still significant at 9 months for *Sutterella* and at 12 months for *Bifidobacterium*. Many associations were observed first at 9 months, and almost all significant associations in the infants delivered via vaginal delivery with antibiotics or C-section were observed after 6 months. After 6 months, *Collinsella* and *Alistipes* were increased in all three delivery groups in infants born to nulliparous mothers compared to multiparous, and *Salmonella* was correspondingly reduced.

### Effect of feeding practices and nutrition on early life gut microbiome

**Fig. 6:**
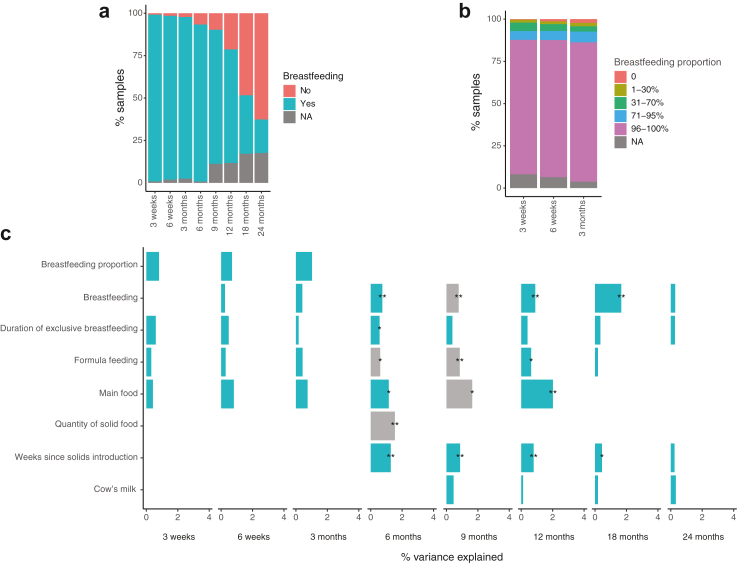
**Infant nutrition and breastfeeding variables by sampling age.** (a) Cohort overview of breastfeeding patterns, (b) proportion of breastfeeding in infant diet until 3 months, and (c) microbial composition variance explained by nutrition variables. Results depict the coefficient of determination (R^2^) from permutational multivariate analysis on Bray–Curtis distances after adjustment with extraction batch and number of processed reads (c). Asterisks mark false discovery rate corrected *P*-values. ∗∗∗: *P* < 0.001, ∗∗: *P* < 0.01, ∗: *P* < 0.05, -: *P* < 0.1, all non-corrected *P*-values <0.05. Grey bars mark uneven beta-dispersion between the categories of a variable. A detailed description of the variables, their definitions and groupings are available in Supplemental File S1.

The impact of early infant nutrition was investigated by examining the breastfeeding practices (breastfeeding and proportion of breastfeeding in the weeks around sampling time, duration of exclusive breastfeeding), time of solid food introduction and its quantity, as well as main food of the infant (breastmilk from breast of bottle, milk or non-milk formula, type of solid food), use of formula or cow's milk adjusting for DNA extraction batch and the number of processed reads. This birth cohort had a very homogenous diet pattern before 6 months, with 95% of the infants being breastfed (Fig. 6a), and 82% exclusively breastfed at 3 months (Fig. 6b). The introduction of solid food took place between weeks 18 and 31 (median = 22 weeks, IQR = 6 weeks). After 9 months, the feeding patterns were more varied, with 67% and 20% of the infants being breastfed at 1 and 2 years old, respectively (Fig. 6a).

Before 6 months, infant nutrition explained a negligible, non-significant proportion of the microbiota variance (Fig. 6c). After 6 months, the effects of continuation of breastfeeding and the timing of introduction of solid foods on the infant microbiota variation became significant, yet, explaining less than 2% of the total variance at 6 months. Breastfed infants had a lower alpha-diversity and taxonomic richness compared to non-breastfed infants at most time points (Supplemental Fig. S10a and b). The absence of breastfeeding was associated with a lower relative abundance of *Lactobacillus* at 3 weeks and from 6 to 18 months, and higher abundance of *e.g., Akkermansia*, *Blautia*, and *Clostridium* clusters XIVa and XI at the same ages (Supplemental Fig. S10c). Additionally, several genera appeared in higher abundances especially in the 18-month sample in non-breastfed infants compared to infants still breastfed. Thus, long-lasting breastfeeding seemed to affect the gut microbiota even when more complex food sources were introduced to the child's diet.

### Effect of health, development, and antibiotic exposures

**Fig. 7:**
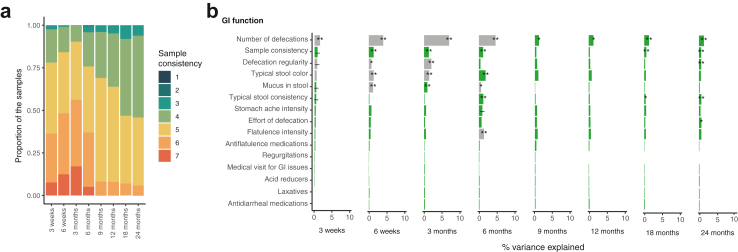
**Variance explained by infant gastrointestinal (GI) function and medication.** (a) Stool consistency of the study samples at different ages recorded by laboratory staff processing the samples, 1 signifying hard separate lumps and 7 watery stool. The proportions were calculated after exclusion of samples without recorded stool consistency. (b) The results depict the coefficient of determination (R^2^) from permutational multivariate analysis on Bray–Curtis distances after adjustment with extraction batch and number of processed reads. Asterisks mark false discovery rate corrected *P*-values. ∗∗∗: *P* < 0.001, ∗∗: *P* < 0.01, ∗: *P* < 0.05, -: *P* < 0.1, all non-corrected *P*-values <0.05. Grey bars mark uneven beta-dispersion between the categories of a variable. A detailed description of the variables, their definitions and groupings are available in Supplemental File S1.

The infant health and development were surveyed through 41 variables covering general health and wellbeing, infections, age-adjusted body mass index (BMI) z-score, motor development, gastrointestinal (GI) function and medication, allergy symptoms, and the fucosyltransferase 2 (FUT2) genotype of the infant^38^ adjusting for DNA extraction batch and the number of processed reads. None of the variables were significantly associated with the infant microbiota variation (Supplemental Fig. S11) except for those related to the infant gut function and stool appearance in particular in the first 6 months of life (Fig. 7b). Specifically, the typical number of defecations per week (classified in three categories by sampling age), the defecation regularity, the typical stool colour, and the presence or absence of mucus in the stool were significantly associated with infant microbiota composition at most time points. Differential abundance testing between defecation frequencies revealed negative associations between *Akkermansia*, *Bacteroides*, *Bifidobacteria*, *Collinsella*, *Eggerthella*, and *Lactobacillus*, and a positive association especially between *Clostridium sensu stricto*, *Haemophilus,* and *Klebsiella* and higher number of defecations throughout the first two years of life (Supplemental Fig. S12a). Higher defecation rate was also associated with a significantly lower genus richness (observed richness) and lower rarity index (log modulo skewness) compared to median and low defecation rates at all time points (Supplemental Fig. S13a–d).

The stool consistency was measured through parents’ estimation of typical stool consistency in the past weeks following the Bristol stool scale, but also by assessing the score of the sample stool in the laboratory at the time of DNA extraction. The concordance between these assessments was low: globally, the two measures are reasonably correlated (Spearman correlation rho = 0.37, *P* = 2.2e-16), but only weakly correlated at each sampling age (rho < 0.1 for most ages). This suggests that the parents assessed typical stool appearance measures the broad stool consistency changes during infancy but does not capture the more subtle changes between each stool. Therefore, these two measures were analysed separately, and the results presented below were produced using the laboratory assessed consistency. The sample stool consistency was found to be significantly associated with the overall microbiota composition at most sampling points (Fig. 7b). Notably, the direction of the association between stool consistency and several individual genera shifted around the 6-month sample (Supplemental Fig. S12b), coinciding with change in the dominant stool form (Fig. 7a). *Bacteroides* and *Clostridium* IV were positively associated with looser stools until 3 months and negatively thereafter, and *Streptococcus* and *Haemophilus* were negatively associated with looser stools in the first months, but positively later on. Notably, the sample Bristol score was significantly associated with the microbiota composition also in parental samples (Supplemental Fig. S14). The parent-reported typical stool consistency was mostly not associated with the microbiota composition (Fig. 7b).

### Effect of family unit, environment, and lifestyle

**Fig. 8:**
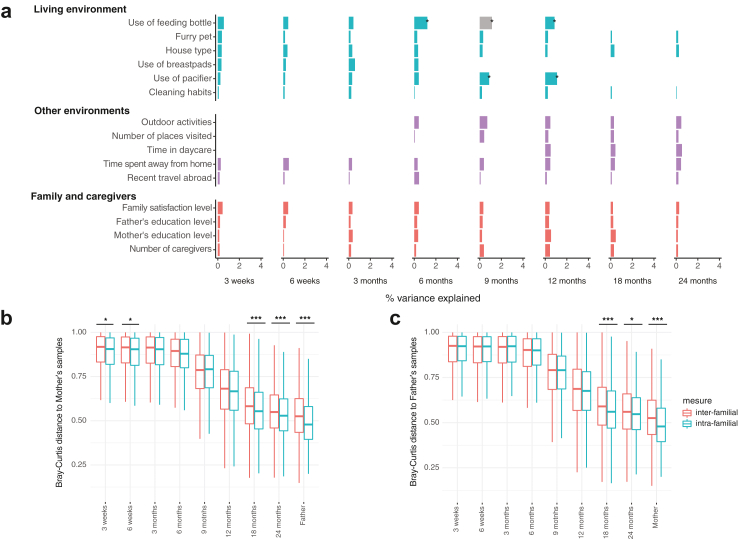
**Environment, lifestyle, and the family unit.** (a) Variance explained by environmental factors and inter- *versus* intra-familial distances to the (b) mothers' and (c) fathers' samples. Results depict the coefficient of determination (R^2^) from permutational multivariate analysis after adjustment with extraction batch and number of processed reads (a). Asterisks mark false discovery rate corrected *P*-values (a) or raw *P*-values (b and c). ∗∗∗: *P* < 0.001, ∗∗: *P* < 0.01, ∗: *P* < 0.05, -: P < 0.1, all non-corrected *P*-values <0.05. Grey bars mark uneven beta-dispersion between the categories of a variable. A detailed description of the variables, their definitions and groupings are available in Supplemental File S1.

To utilise the availability of gut microbiota profiles of infant-mother-father triads, we assessed their compositional distance within and between families. A significant effect of the family unit was observed: Bray–Curtis dissimilarity was significantly lower between mother-infant dyads than between families in neonatal phase (3 and 6 weeks), and 18 months onwards (Fig. 8b). This was true also for the infant-father dyads from 18 months onwards as well as the spouses (Fig. 8c). The microbiota of mothers and fathers resembled more their own spouse than that of the other same sex parents.

The impact of environmental factors on infant gut microbiota variation was assessed through 15 variables on care practices and household related factors (use of a pacifier, feeding bottle, or breast pads, pets, type of housing, cleaning habits), as well as external exposures (time spent away from home, travel abroad, time spent outdoors, number of places visited, day care) adjusting for DNA extraction batch and the number of processed reads. The number of caregivers, parents’ level of education and satisfaction of family life were also included. The effect size of most of the individual environmental variables accounted for less than 1% of the infant gut microbiota variance (Fig. 8a), with the notable exception of the use of a feeding bottle (proxy for formula use) between 6 and 12 months and pacifier at 9 and 12 months.

We also examined the associations between parental gut microbiota variation and 27 variables describing their health, diet, socioeconomic status, and lifestyle (Supplemental Fig. S14). Beyond the sample Bristol score (mothers and both parents pooled 0.3%) and BMI (fathers 0.6%, mothers 0.4%, parents pooled 0.3%), significant associations were observed with allergic diseases (parents pooled 0.4%), maternal probiotic (0.7%) and fatty acid supplement use (0.3%), and parity (0.4%).

### Cumulative variance explained by significant exposures in infant microbiota community

Finally, we assessed the total cumulative variation explained by the variables investigated in this study. Cumulative models were constructed with the biological variables and interactions with a significant impact on the infant microbiota at each age adjusting for DNA extraction batch and the number of processed reads. After backwards selection of the variables, the models included 9 to 12 biological variables and explained 7.1%–19% of the microbiota variation (Supplemental Fig. S15, Supplemental File S2), surpassing the cumulative technical variation (Supplemental Fig. S5) in all ages. The maximum variance explained by this approach was for the 3 months samples with 19% of the variation explained. Globally, the total variation explained by the cumulative models were lower in later time points. All models included the three-level delivery variable (delivery mode and antibiotic prophylaxis), which explained 8% of the microbial variation at 3 weeks, but slowly decreased in importance to only 0.3% at 2 years. Variables describing GI function remained important throughout the 2 years. The importance of nutrition peaked at 6–9 months after the introduction of solids, but nutrition variables remained in most models after model selection signifying their consistent importance.

To assess the effect of infant age and the individual variation, we constructed a model with all the sampling points with the most important variables identified in previous analyses across different ages adjusting with the number of processed reads (Supplemental Fig. S16). As expected, individual variation was the strongest effector, followed by infant age. The two variables accounted for 36% and 12%, respectively, out of the total 50% of the variation explained by the biological variables in the model. The remaining variables were consistent with the previous time-stratified models and mainly accounted for GI function (number of defecations, DNA yield, stool consistency, stool colour), but also breastfeeding and infant BMI z-score.

## Discussion

The aim of this study was to assess the relative and cumulative temporal importance of different early life exposures explaining the infant gut microbiota community variation, utilising frequent microbiota sampling, detailed records of technical variates, and rich metadata available in the Finnish longitudinal HELMi birth cohort.^23^ We systematically explored the associations between 109 metadata variables and infant gut microbiota composition during the first two years of life, and 36 and 26 variables in mothers and fathers spot samples, respectively, in a dataset of 7657 samples from 985 families. We first identified the most important technical variables in this cohort and adjusted the biological variable analyses with them to diminish their confounding effect. These non-causal confounders can be particularly important to control for, as they may mask or bias true biological associations of interest between exposures and microbiota composition.

Despite dedicated efforts to collect and process samples within a study in the same way, several possible sources of technical variation still exist^39^ and can exceed the effect of biological variables.^40^ Despite adhering to a validated^20^^,^^41^ mechanical DNA extraction method with a standardised operating procedure, we observed a significant impact from extraction batch of up to 10% of total variance in all sample types, while other technical variables, such as storage time, had a very limited impact. Few large-scale infant microbiota studies have assessed the batch effect and attempted to account for technical effects,^2^^,^^42^ *e.g.*, Crane et al.^22^ report an effect size of 5%–8% from sequencing batch, in a dataset of around 70 infants per age group. Our cumulative effect of the technical variables ranged from 6.8 to 15% in the 6378 infant samples and 11–13% in the 1279 parental samples. The higher effect size in early infant samples was mainly due to the larger number of extraction batches at those time points. The higher percentage obtained for parental samples is at least partly explained by the smaller beta-diversity of adult samples; when the beta-diversity is lower, a technical difference can proportionally have a larger impact. We also assessed the effect of different beta-diversity distance methods and the effect of using different taxonomic levels. Although our results were highly reproducible regardless of method, the choice can impact on the results attained,^43^ warranting future studies to investigate methods for their ability to robustly identify biological versus technical variation across different cohorts. Bray–Curtis distance was chosen for this study due to its wide use and comparability to other studies. We recapitulate the previously stated^39^^,^^44^ urge to study and transparently report effect sizes for technical covariates in microbiome studies to attain more reliable, biologically accurate results.

The effect of birth mode on infant gut microbiota development is well-established, and typically seen for months after birth.^4^, ^5^, ^6^, ^7^ In the HELMi cohort, the effect of delivery mode on the global infant microbiota composition was significant until 12 months. We also examined the differences between intrapartum antibiotic prophylaxis in addition to birth mode, as in the subset of this cohort we previously observed that birth mode and intrapartum antibiotic had distinct effects using quantitative profiling of the infant gut microbiota.^9^ We recapitulated the stronger effect of birth mode compared to antibiotics, but saw a clear reduction in *Bacteroides* in vaginally delivered intrapartum antibiotic-exposed infants, not seen in our previous study.^9^ This is likely due to the nature of relative abundances used in this study compared to the absolute abundances in the previous study: an increase of one taxon will reduce the proportion of other taxa with no change in their absolute numbers. Many of the perinatal factors were associated with delivery mode, implying that mother and infant microbiota studies should consider whether to preferentially stratify rather than adjust for birth mode not to unintentionally remove the effect of many other perinatal effects of potential interest (*e.g.*, discharge from the maternity ward is more likely to be delayed after C-section delivery).

Parity, reflecting mother's reproductive history and the presence of older siblings, was consistently associated with the infant gut microbiota composition across the two-year follow-up, typically explaining at least 1% of the variation. Parity was also associated with maternal gut microbiota composition. The effect of parity or older siblings on infant microbiota has been observed previously^2^^,^^17^^,^^18^^,^^45^ and it is mainly believed to reflect the added microbial exposures from the older siblings. Recently, infants with older siblings were found to have a more mature gut microbiota at 1 year, which in turn was associated with a lower prevalence of food allergy.^46^ However, recent papers indicate that parity modulates the maternal gut^47^^,^^48^ and vaginal microbiota^49^ during pregnancy, and thus could affect the microbiome transferred to the offspring. Analyses stratified by delivery mode and intrapartum antibiotics revealed clear birth mode-dependent temporal patterns, suggesting early effects seen mainly in infants delivered vaginally without antibiotics are likely of maternal origin transmitted during the delivery. On the other hand, the effect of parity was stronger in the C-section delivered or intrapartum antibiotic-exposed infants than the unexposed vaginally delivered infants at 6 and 9 months of age, possibly indicating that the presence of older siblings could mitigate some of the effects from C-section delivery or antibiotics on the early colonisation.

Stool consistency, a good alternative to the difficult to measure transit time,^50^ was one variable most consistently associated with the gut microbiota across different ages, with a coefficient of determination of 0.5–1%, and was also significant in the adults. Interestingly, we saw a shift in the direction of some bacterial associations with stool consistency around the 6-month sampling, reflecting the change in dominant stool form as a result of the introduction of solid foods. Beller *et al.*^1^ found stool consistency to be associated with the gut microbiota composition in eight closely followed infants. We are not aware of further studies on infant stool consistency and microbiota in the general population, only in functional gastrointestinal orders^51^ and as intervention outcomes.^52^^,^^53^ However, Bristol score is a common measure of stool consistency in adults with a well-established link to gut microbiota composition,^54^ and has been identified to explain 5% of the total microbiota variation in adult gut microbiota,^55^ indicating that the association between gut microbiota and stool consistency is important as suggested also by our previous study.^9^ Future work should also carefully assess bidirectionality as it is possible that stool composition is a consequence, not a cause, of microbiota composition. Importantly, the laboratory- and parent-reported infant stool consistencies had low correlation and the former was more strongly associated with microbiota composition. This may reflect the objectivity of the staff performing the assessment and variation in stool consistency between passings of stool. An age-validated modified score may help parents to more reliably evaluate both diaper^56^ and potty-sampled specimens,^57^ and would be preferable in future infant microbiota studies. Infant GI function and stool appearance are also highly affected by age and feeding habits,^58^ and in consequence the microbiota changes associated with these variables are probably tightly tied in with breastfeeding.

Despite the age-driven diversification of the gut microbiota, we saw a steady increase of the total variance explained by the biological variables from 3 weeks to 3–6 months, peaking at 19%, and decreasing down to around 7% by 24 months. At 3–6 months, the inter-individual differences in the gut microbiota composition had reduced and at the same time there was more diversity in the metadata *e.g.*, diet. The different GI variables, specifically stool consistency, were the most consistently associated throughout the two-year follow-up whereas the effects of delivery diminished, and diet increased as the child got older. Interestingly, infant pacifier use remained in the 12-month model after backward selection and was significant at 9 and 12 months in the individual PERMANOVAs. A previous study identified pacifier use, and more specifically pacifier antiseptic use at 6 months, to be associated with a higher likelihood of food allergy development,^59^ alluding to a potential link between pacifier use, infant microbiota, and allergy development. We are not aware of other studies quantifying the total variance explained of infant microbiota at different ages, but in adult samples, a large cohort was able to explain 8% of the total microbiota variation.^55^ Across the pooled infant samples from week three to month 24, infant identity and age together accounted for 48 percentage points of the total 50% of variance explained. In previous studies, similar numbers for longitudinal analyses have been attained in smaller cohort sizes.^1^^,^^17^ Despite the vast collection of data, a large share of the microbiota variation remains unexplained even in this cohort: *e.g.*, the effect of genetics (apart from FUT2 genotype) was not analysed here and overall remains largely unexplored for early life microbiota. The difficulties to characterise gut microbiota variation and possible underlying reasons have been discussed in more detail by Schmidt *et al*.^60^

In this study, infants' FUT2 secretor genotype, infant probiotic use or the medications tested, including antibiotics, were not statistically significantly associated with gut microbiota beta-diversity. It is important to note that the majority of the families participating in the HELMi cohort lived in the capital region of Finland and had homogenous lifestyles. All the infants were born term, thus no differences due to prematurity were investigated, and the infants were generally healthy; due to the small sample sizes we excluded other chronic diseases except allergies. The cohort was also characterised by high breastfeeding rates and late cessation of breastfeeding. In consequence, some previously established effects were missing in our results; *e.g.*, infant microbiota variation associated with feeding practices only from 6 months onwards, after introduction of solids into the infant's diet. Similarly in a Dutch cohort with high breastfeeding rates, the effects sizes of breastfeeding ranged from 1 to 2%.^61^ Interestingly, we saw an effect from continued breastfeeding lasting until 18 months, corroborating previous research about the importance of continued breastfeeding on the infant gut microbiota even after the introduction of solids.^13^ Similarly to two other recent studies,^2^^,^^61^ we found no significant effects from postnatal antibiotics on the genus level bacterial composition probably due to the sparsity and scatterness of the antibiotic courses and the statistical methods. No effects of probiotics could either be observed on the infant gut microbiota variation, possibly due to the high usage rate (70% by age of 3 months),^7^^,^^9^^,^^23^ simplified variables, and the statistical methods used. These results highlight the need to interpret and compare results in the context of each cohort's characteristics and homogeneity. In this paper, we could not address the difference in effect of parity depending on the age of the older siblings, but we consider this an important future study question due to difference in exposures of children of different ages. Further, the analyses in this study were performed on genus level data and mostly on beta-diversity. Many biologically relevant results can happen on lower taxomical levels and not show up on beta-diversity level. The results are also associative and any causal relationships are based on previous knowledge.

In summary, our results highlight the importance of considering technical variables in the planning stages, the choice of analysis methods and confounders, and in the interpretations of results, especially in large datasets. Throughout the follow-up period, variables explaining GI function showed significant associations with the gut microbiota composition. More research is needed in the relationship between infant GI function and gut microbiota to better understand the causality between these variables. Importantly, we also saw a delivery mode and intrapartum antibiotic dependent effect from parity/older siblings, highlighting the importance of considering delivery mode-stratified analysis over adjustment in infant gut microbiota studies. The effect of family unit was emphasised in our results, both in infant and parental faecal samples. Our results will guide future research in exploring the potential confounders or stratification in the analysis, and to better understand possible external determinants such as early life nutrition at different stages of the gut microbiota development.

## Contributors

WMdV and AS conceptualised the HELMi cohort and together with K-LK and KK designed it. AS, KK, RJ and AJP designed the current study. Generation of data ED and analysis and interpretation of data RJ, AJP, XW, and AS. Writing the first version of the manuscript RJ and AJP. All authors read, edited, revised, and approved the final version of the manuscript.

## Data sharing statement

Sequencing data are accessible in ENA along with limited metadata (Study ID: PRJEB55243 https://www.ebi.ac.uk/ena/browser/view/PRJEB55243). Additional data can be obtained from the corresponding author upon reasonable request.

## Declaration of interests

The authors declare no competing interests.
