# Supplementary material for: Sources of gut microbiota variation in a large longitudinal Finnish infant cohort
Source: eBioMedicine. 2023 Jul 1;94:104695. doi: 10.1016/j.ebiom.2023.104695 (PMC10328818; doi:10.1016/j.ebiom.2023.104695)
Supplement: Supplemental Figures and Table [file mmc3.pdf]

# SUPPLEMENTAL MATERIAL

## Table of contents


Supplemental results

Supplemental tables

- **Supplemental Table 1.** Overview of the study cohort characteristics

Supplemental figures

- **Supplemental Figure 1.** Associations between delivery mode and (a) other delivery variables, (b) parental, (c) breastfeeding and nutritional variables, and (d) technical variables
- **Supplemental Figure 2.** Composition and diversity of faecal microbiota of infants from 3 weeks to 2 years of age and their parents
- **Supplemental Figure 3.** Associations between DNA yield and relative abundances of different genera by sampling age
- **Supplemental Figure 4.** Associations between sequencing platform and (a) evenness, (b) rarity, and (c) relative abundances of different genera by sampling age
- **Supplemental Figure 5.** Cumulative microbiota variation explained by technical variables measured by sampling age and parental samples
- **Supplemental Figure 6.** Differences between different distance methods and taxonomic levels in the effect sizes of technical variables by sampling age and parental samples
- **Supplemental Figure 7.** Variance explained in the infant microbiota by parental and pregnancy variables by sampling age

- **Supplemental Figure 8.** Effect of birth mode and intrapartum antibiotics on the (a) relative abundances of different genera, (b) alpha-diversity, and (c) richness by sampling age
- **Supplemental Figure 9.** Effect of parity on (a) alpha-diversity, (b) richness, (c) evenness, and (d) rarity by sampling age
- **Supplemental Figure 10.** Associations between breastfeeding and (a) alpha-diversity, (b) richness, and (c) relative abundances of different genera by sampling age
- **Supplemental Figure 11.** Variance explained by infant health and development by sampling age
- **Supplemental Figure 12.** Associations between (a) defecation rate and (b stool consistency and relative abundances of different genera by sampling age
- **Supplemental Figure 13.** Associations between defecation rate and (a) alpha-diversity, (b) richness, (c) evenness, and (d) rarity by sampling age
- **Supplemental Figure 14.** Variance explained in mothers' and fathers' samples and parental samples pooled together
- **Supplemental Figure 15.** Total bacterial variation explained by biological variable groups by sampling age
- **Supplementary Figure 16.** Total bacterial variation explained in all infant samples from the first 2 years of life

## Supplemental files

- **Supplemental File 1.** Variable definitions and characteristics included in this study.

- **Supplemental File 2.** Permutational multivariate analysis by sampling age for the most important biological variables

# References

# Supplemental results

## Cohort overview

As expected, the birth mode (vaginal delivery or C-section) was associated to several other perinatal variables (gestational age, intrapartum antibiotic exposure, extra milk received at the hospital, intensive care at birth, age of the first skin-to-skin contact, age of the infant at discharge from hospital) (Supplemental Fig. S1a), as well as some maternal and paternal factors (age, pre-pregnancy BMI, and parity) (Fig. S1b). Importantly, the delivery mode was also associated with infant and breastfeeding factors such as the length of exclusive breastfeeding and the age of first solid food introduction (Fig. S1c). Finally, an association between the birth mode and some technical variables in our study was observed (DNA extraction batch and sequencing run) (Fig. S1d) due to the overrepresentation of C-section samples in the first sequencing runs.[1]

## Impact of technical variables in faecal microbiota variation in a large cohort of infants and their parents

The total length of the sample storage, the length of storage at home (-20 °C) or at the laboratory (-80 °C) did not have a consistent nor significant impact on the DNA extraction yield nor alpha-diversity measures. The DNA yield was significantly affected by the extractor (Kruskal-Wallis, $P<0.05$, stratified by sample type (infant samples by age and parent samples separately)). However, DNA yield was not significantly associated with alpha-diversity, richness, evenness or rarity in any sample type (Spearman $R^2$ |0.3|, samples stratified by sequencing platform), indicating that also lower DNA yields were representative in terms of taxonomic coverage at least with the mechanical lysis protocol used in this study. The result is in line with the fact that while faecal DNA yield is influenced by technical variables such as the method of extraction, it is also

influenced by biological factors such as the stool consistency,[2] age, and faecal transit time. Using differential abundance testing, we further investigated the effect of DNA yield on the microbiota composition. Several low-abundant genera, in particular *Streptococcus*, *Staphylococcus*, *Rothia*, and *Gemella*, were negatively associated with faecal DNA yield during the first 6-9 months (Fig. S3). While these taxa are commonly reported for infant gut microbiota[3] they also represent the core taxa of infant oral microbiota[4] and are common contaminants in low-biomass microbiome studies,[5] making it difficult to assign their overrepresentation in samples with low DNA yield reliably either to a carry-over from the upper gastrointestinal track or external contamination. Also, dominant taxa such as *Bacteroides*, *Bifidobacteria*, and adult-type Firmicutes were found to vary according to the DNA yield, mainly at 24 months and in adult samples (Fig. S3).

# Supplemental tables

**Table S1: Overview of the study cohort characteristics.** Background and early exposure characteristics were stratified by mode of delivery (C-section or vaginally delivered infants). Continuous variables are shown as median with interquartile ranges (IQR). Categorical variables are shown in absolute numbers with percentages (%) of the birth groups. The number of infants with missing values is indicated in the column "Number of missing values" and is shown as the infant counts and percentage of the complete cohort.

| | | C-section delivered | Vaginally delivered | Complete cohort | Number of missing value (% cohort) |
|---|---|---|---|---|---|
| | n | 161 | 824 | 985 | |
| | Sex female (%) | 81 (50.3%) | 411 (49.9%) | 492 (49.8%) | 0 (0%) |
| **Samples collection** | | | | | |
| | All time points (%) | 53 (32.9%) | 301 (36.5%) | 354 (36%) | |
| | Average number of samples per infant | 6 | 6 | 6 | |
| **Peri-natal variables** | | | | | |
| | Intrapartum antibiotics (%) | 161 (100%) | 193 (23.4%) | 354 (36%) | 1 (0.1%) |
| | Ruptured membranes in hours, median (IQR) | 0.01 (19.6) | 5.73 (12.1) | 5.45 (13.4) | 78 (8%) |
| | Received hospital care after birth (%) | 27 (16.8%) | 56 (6.8%) | 83 (8.4%) | 0 (0%) |
| **Family variables** | | | | | |
| | BMI pre-pregnancy, median (IQR) | 23.9 (4.6) | 22.5 (4) | 22.7 (4) | 8 (0.8%) |
| | Mother has previous deliveries (%) | 60 (37.3%) | 449 (54.5%) | 509 (51.7%) | 0 (0%) |
| | Family has one or more pets (%) | 58 (36%) | 292 (35.5%) | 350 (35.5%) | 0 (0%) |
| | Mother received a university level education (%) | 137 (85.1%) | 739 (89.7%) | 876 (88.9%) | 0 (0%) |
| **Infant variables** | | | | | |
| | Length of exclusive breastfeeding in weeks, median (IQR) | 8 (18) | 18 (19) | 15 (20) | 135 (13.7%) |
| | Age of first solids in weeks, median (IQR) | 20 (6) | 22 (6) | 22 (6) | 9 (1%) |
| | Post-natal antibiotics during the two years (%) | 79 (49%) | 356 (43.2%) | 435 (44%) | 0 (0%) |

# Supplemental figures

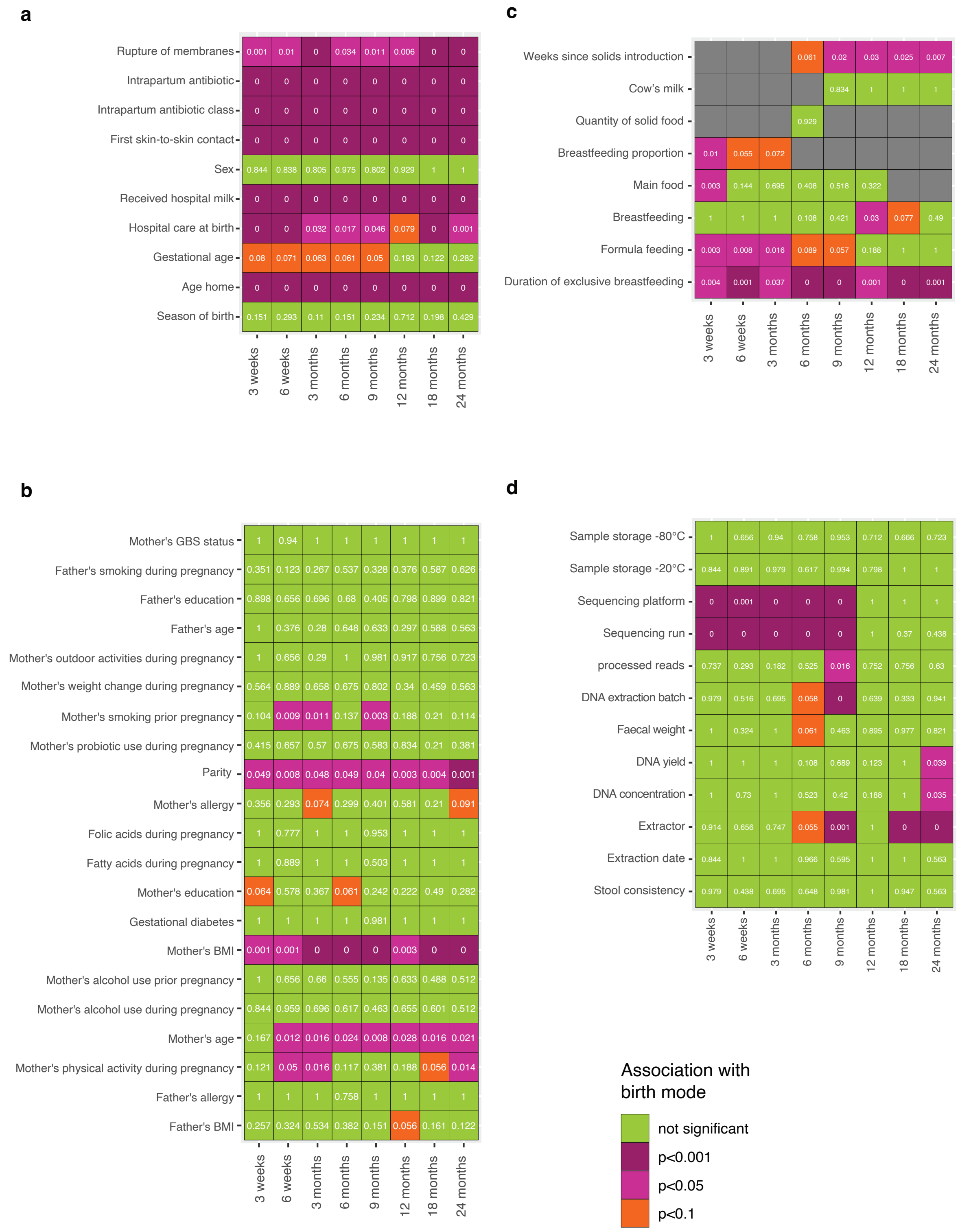


**Fig. S1 Associations between delivery mode and (a) other delivery variables, (b) parental, (c) breastfeeding and nutritional variables, and (d) technical variables.** The association between the mode of delivery (vaginal or C-section) and other variables in the HELMi cohort were assessed at each sampling point independently using Wilcoxon signed-rank test for numeric variables and Fisher exact test for categorical and Boolean variables. The *P*-values were FDR adjusted to correct for multiple comparisons. A detailed description of the variables, their definitions and groupings are available in Supplemental File 1.

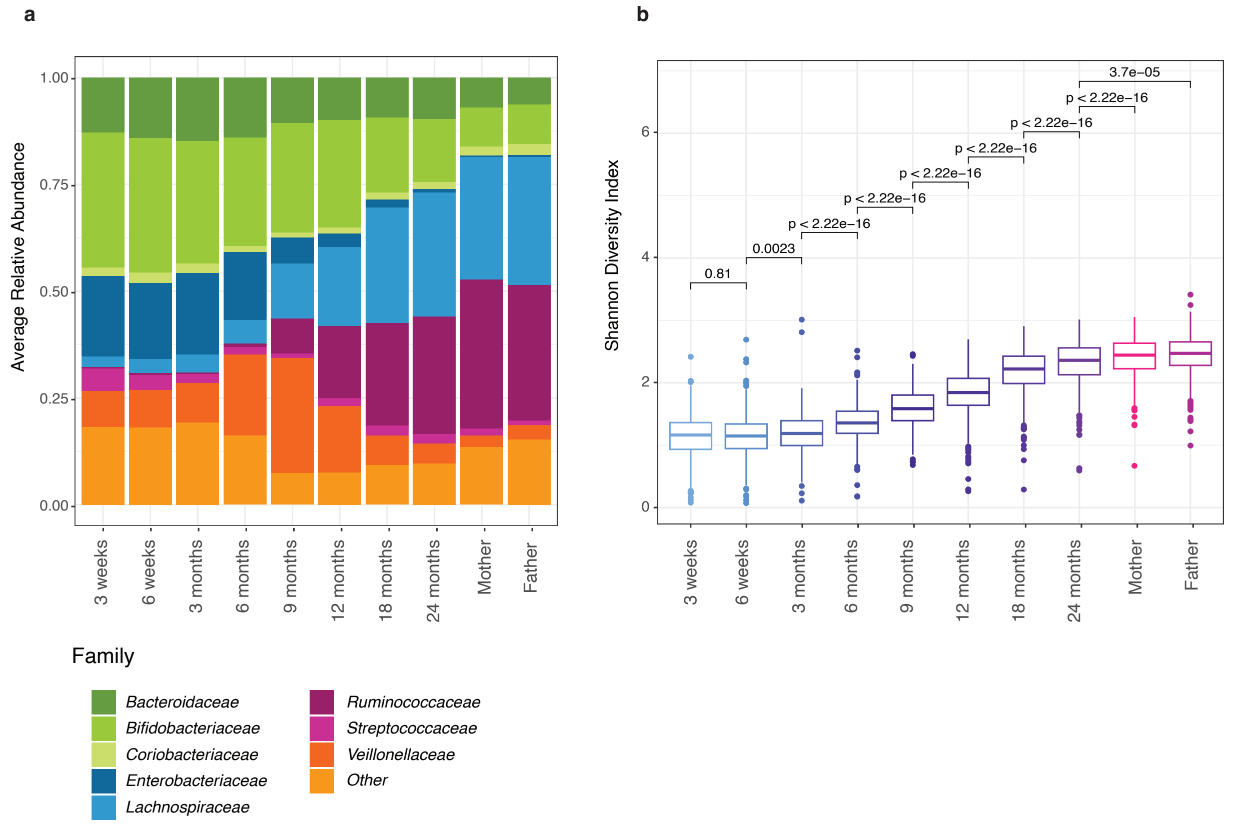


**Fig. S2 Composition and diversity of faecal microbiota of infants from 3 weeks to 2 years of age and their parents.** (a) Average taxonomic composition of the samples. Counts were aggregated at the family level, and taxa accounting for less than 5% of the relative abundance and with a prevalence below 10% were grouped as "Other". (b) Alpha-diversity of the samples. Shannon diversity index was computed for all samples on taxonomic counts aggregated at the genus level. Comparison between sample groups were assessed using Wilcoxon signed-rank test. Similar results were observed after rarefying the counts (data not shown).

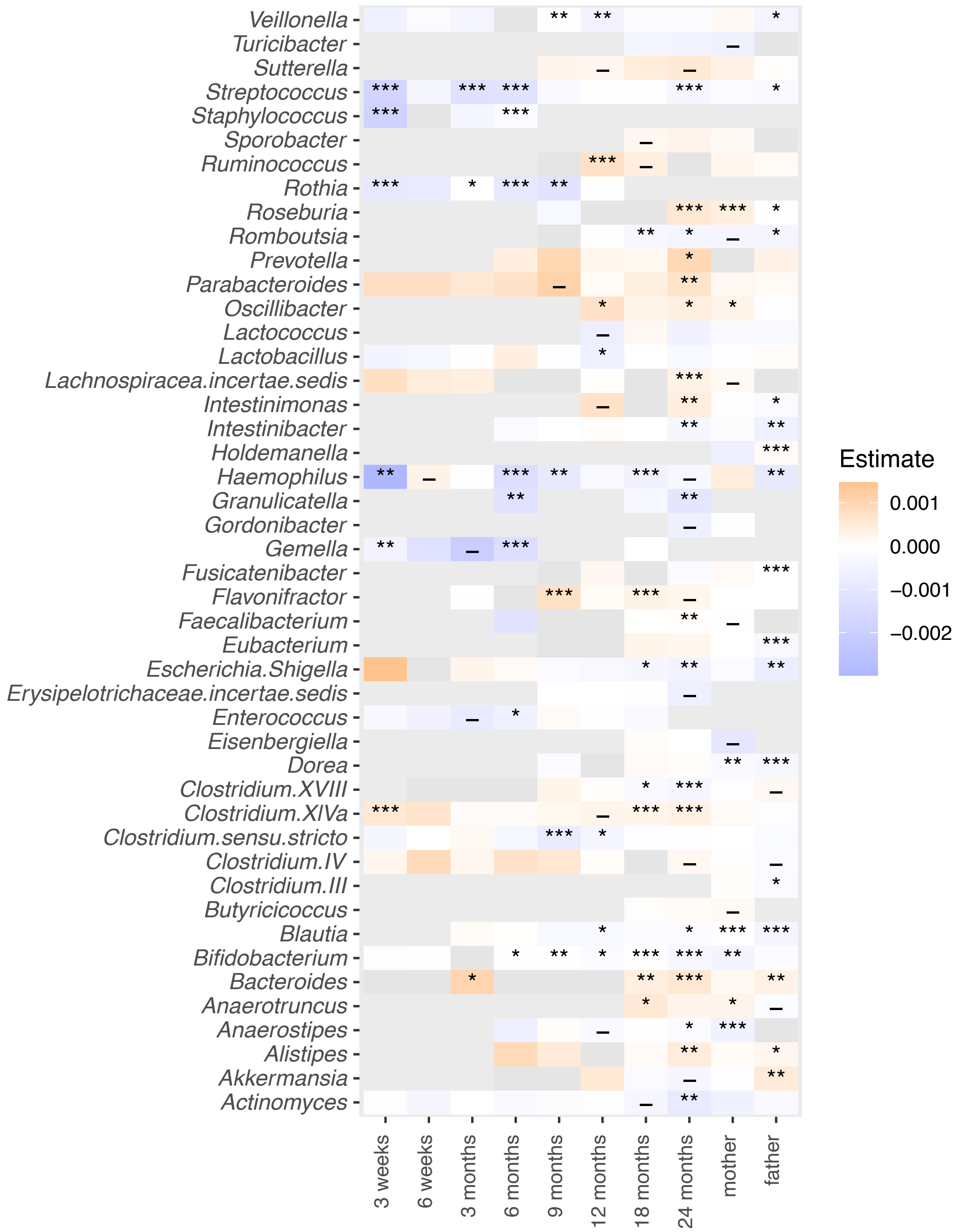


**Fig. S3 Associations between DNA yield and relative abundances of different genera by sampling age.** Orange colour implies a positive association with increasing DNA yield and purple a negative association. Analysis adjusted for sample stool consistency. Asterisks mark false discovery rate corrected *P*-values. ***: *P*<0.001, **: *P*<0.01, *: *P*<0.05, -: *P*<0.1, all non-corrected *P*-values <0.05. A detailed description of the variables, their definitions and groupings are available in Supplemental File 1.

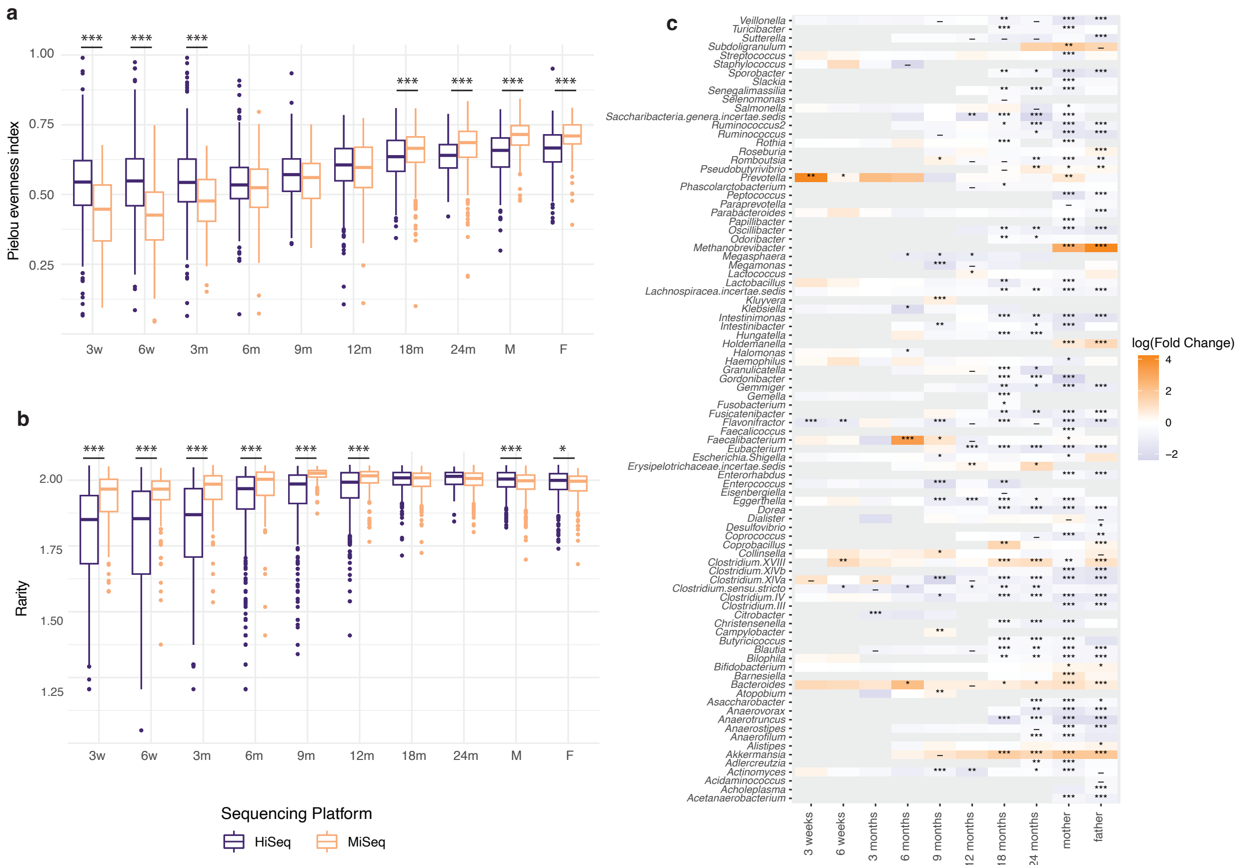


**Fig. S4 Associations between sequencing platform and (a) evenness, (b) rarity, and (c) relative abundances of different genera by sampling age.** MiSeq platform is used as the reference, indicating orange colours to symbolise positive and purple negative associations with HiSeq. Rarefaction to the lowest number of processed reads at each time point was performed before computing the evenness and rarity (a and b). Asterisks mark raw *P*-values (a and b) or false discovery rate corrected *P*-values (c). ***: *P*<0.001, **: *P*<0.01, *: *P*<0.05, -: *P*<0.1, all non-corrected *P*-values <0.05. A detailed description of the variables, their definitions and groupings are available in Supplemental File 1. w=weeks, m=month, F=father, M=mother.

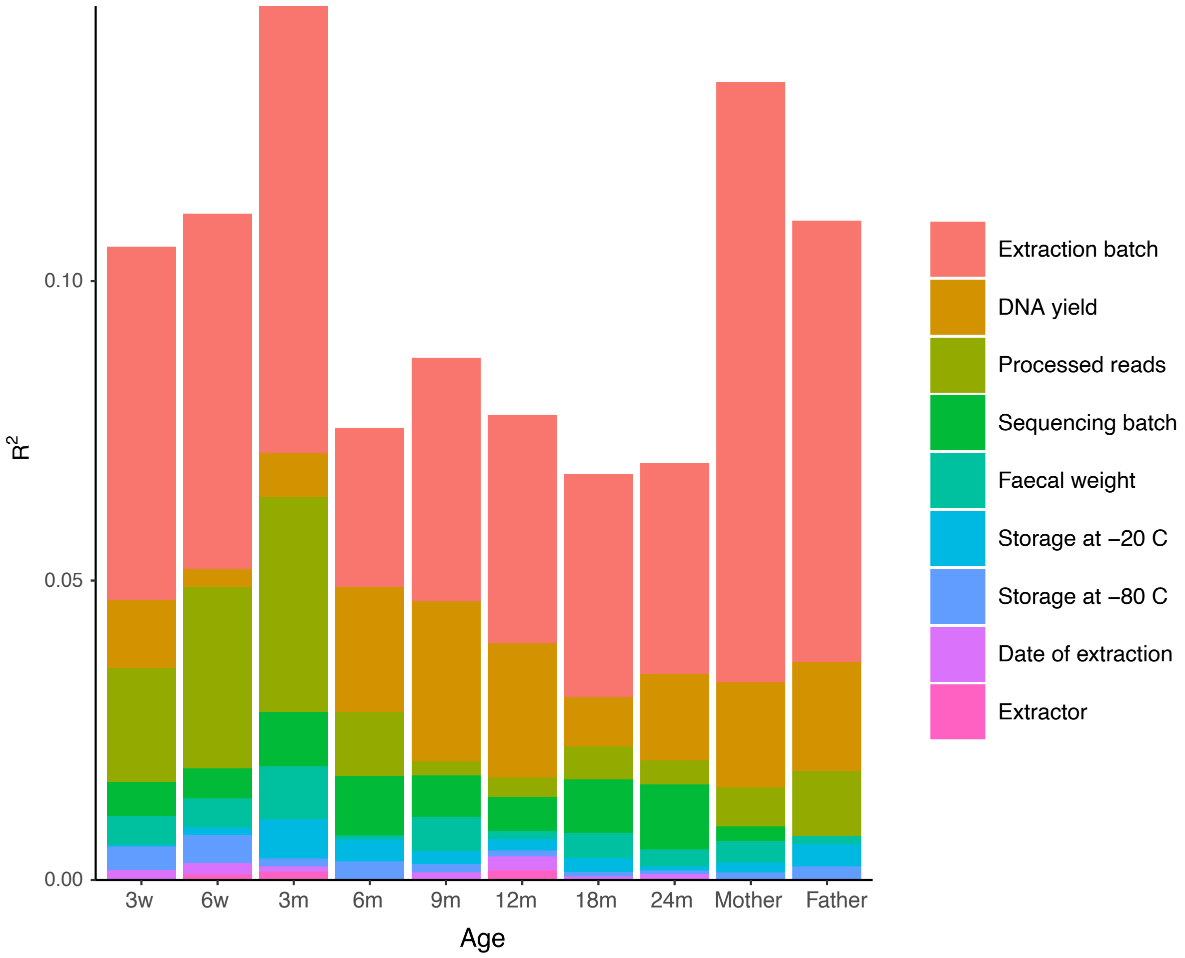


**Fig. S5 Cumulative microbiota variation explained by technical variables measured by sampling age and parental samples.** Permutational multivariate models by age and parental samples. A detailed description of the variables, their definitions and groupings are available in Supplemental File 1. w=week, m=month.

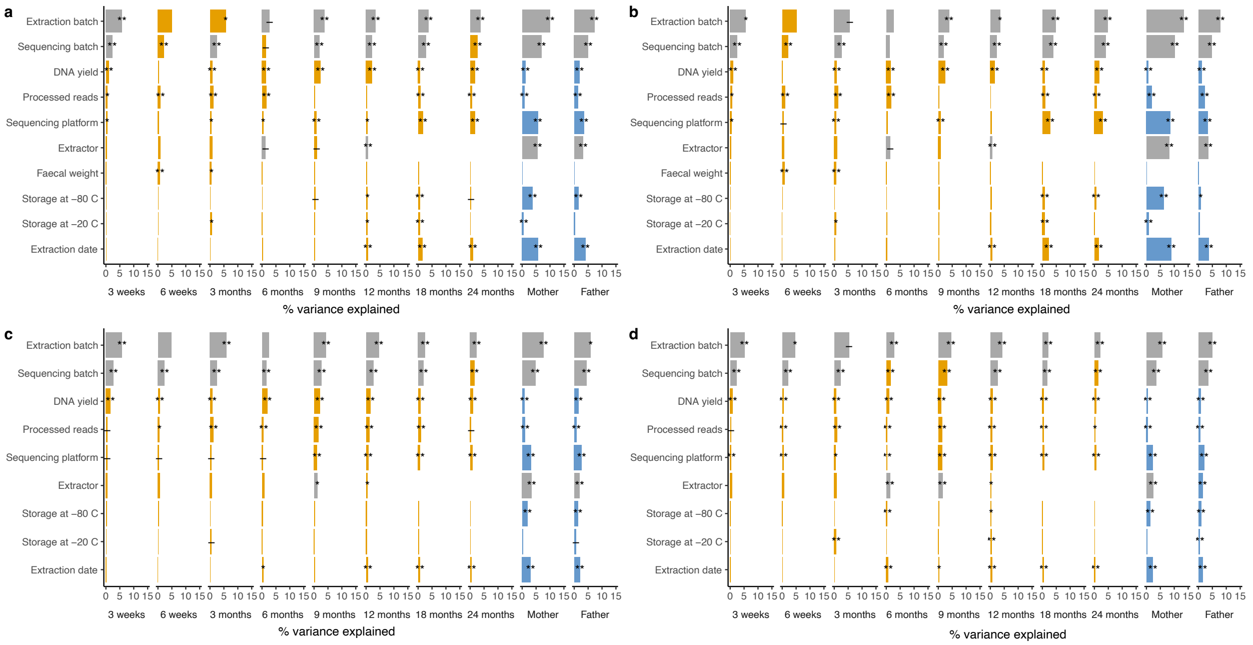


**Fig. S6 Differences between different distance methods and taxonomic levels in the effect sizes of technical variables by sampling age and parental samples.** Permutational multivariate analysis performed with Bray-Curtis distance on (a) genus level and (b) family level bacterial compositions, or with (c) Pearson's or (c) Aitchison distance on genus level compositions. Asterisks mark false discovery rate corrected P-values. ***: $P<0.001$, **: $P<0.01$, *: $P<0.05$, -: $P<0.1$, all non-corrected $P$-values <0.05. Grey bars mark uneven beta-dispersion between the categories of a variable. A detailed description of the variables, their definitions and groupings are available in Supplemental File 1.

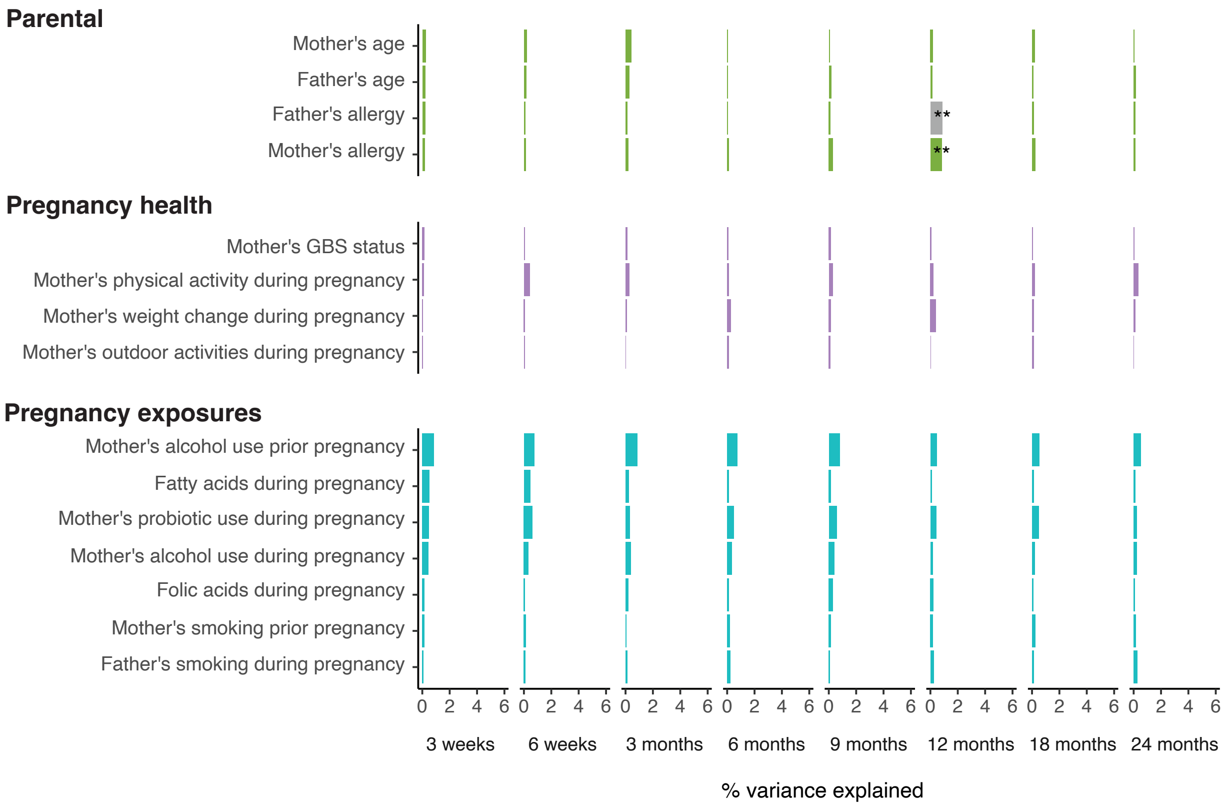


**Fig. S7 Variance explained in the infant microbiota by parental and pregnancy variables by sampling age.** Results depict the coefficient of determination ($R^2$) from permutational multivariate analysis on Bray-Curtis distances after adjustment with extraction batch and number of reads. Asterisks mark false discovery rate corrected *P*-values. ***: *P*<0.001, **: *P*<0.01, *: *P*<0.05, -: *P*<0.1, all non-corrected *P*-values <0.05. Grey bars mark uneven beta-dispersion between the categories of a variable. A detailed description of the variables, their definitions and groupings are available in Supplemental File 1.

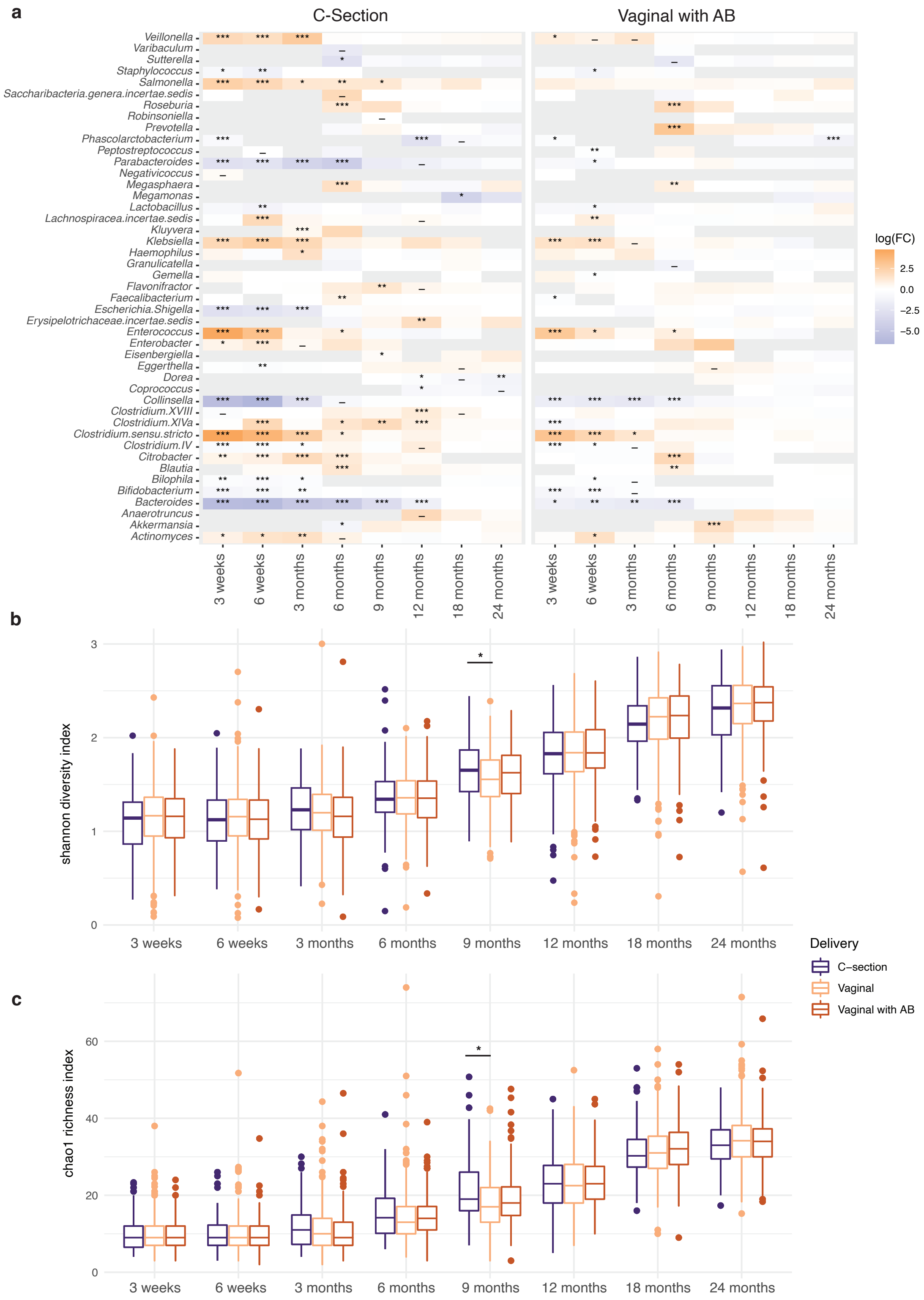


**Fig. S8 Effect of birth mode and intrapartum antibiotics on the (a) relative abundances of different genera, (b) alpha-diversity, and (c) richness by sampling age.** Vaginal delivery without intrapartum antibiotics used as reference in the analyses *P*-values for (a). Asterisks mark false discovery rate corrected *P*-values. *P*<0.001, **: *P*<0.01, *: *P*<0.05, -: *P*<0.1, all non-corrected *P*-values <0.05. A detailed description of the variables, their definitions and groupings are available in Supplemental File 1. FC=fold change, AB=antibiotic.

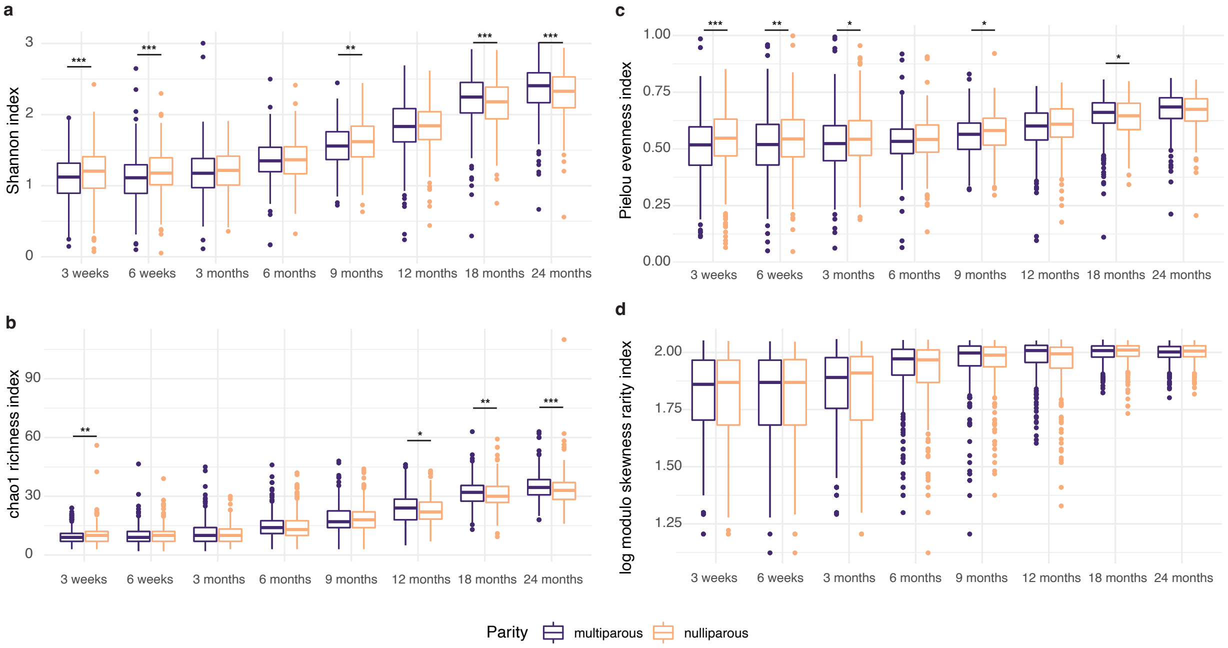


**Fig. S9 Effect of parity on (a) alpha-diversity, (b) richness, (c) evenness, and (d) rarity by sampling age.** Asterisks mark *P*-values. ***: *P*<0.001, **: *P*<0.01, *: *P*<0.05. A detailed description of the variables, their definitions and groupings are available in Supplemental File 1.

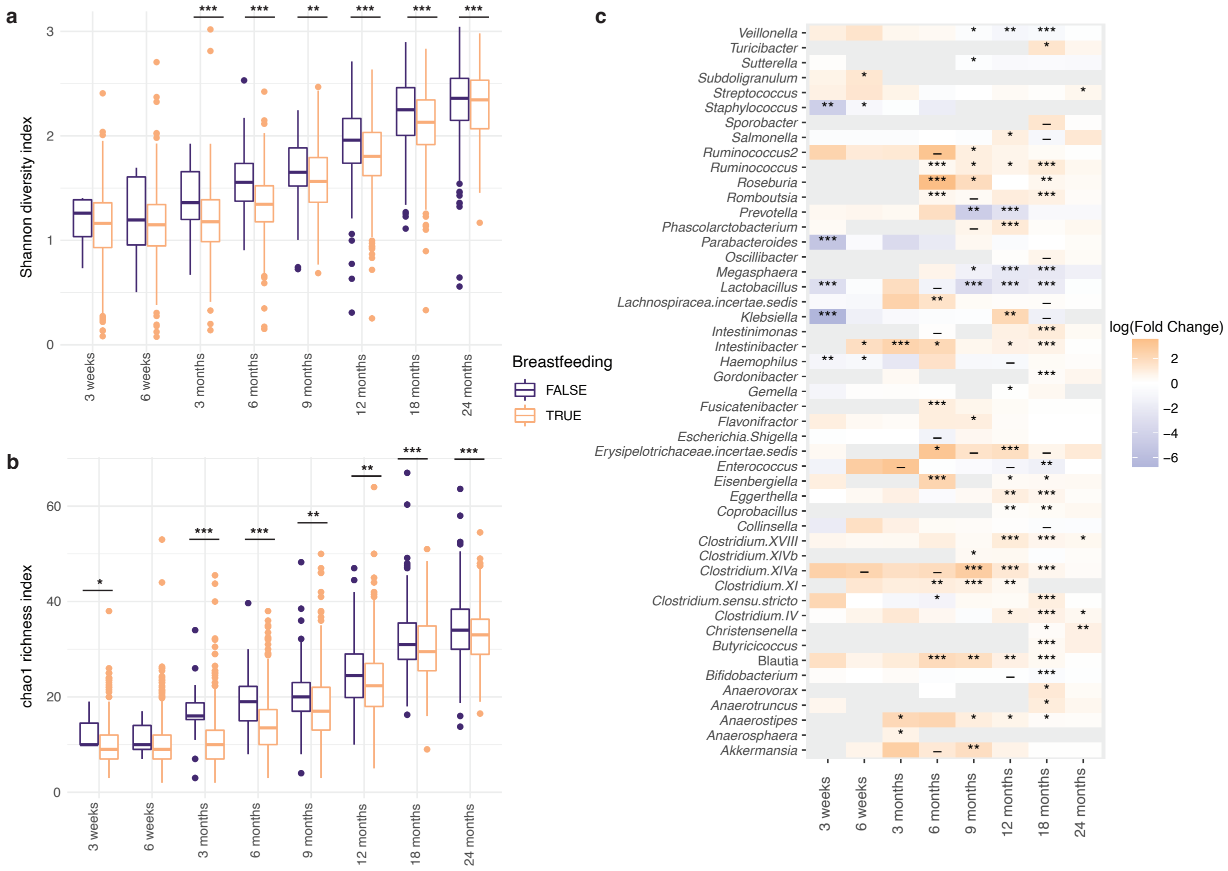


**Fig. S10 Associations between breastfeeding and (a) alpha-diversity, (b) richness, and (c) relative abundances of different genera by sampling age.** Ongoing breastfeeding is used as reference (c). Asterisks mark raw *P*-values (a and b) or false discovery rate corrected *P*-values (c). ***: *P*<0.001, **: *P*<0.01, *: *P*<0.05, -: P<0.1, all non-corrected *P*-values <0.05. A detailed description of the variables, their definitions and groupings are available in Supplemental File 1.

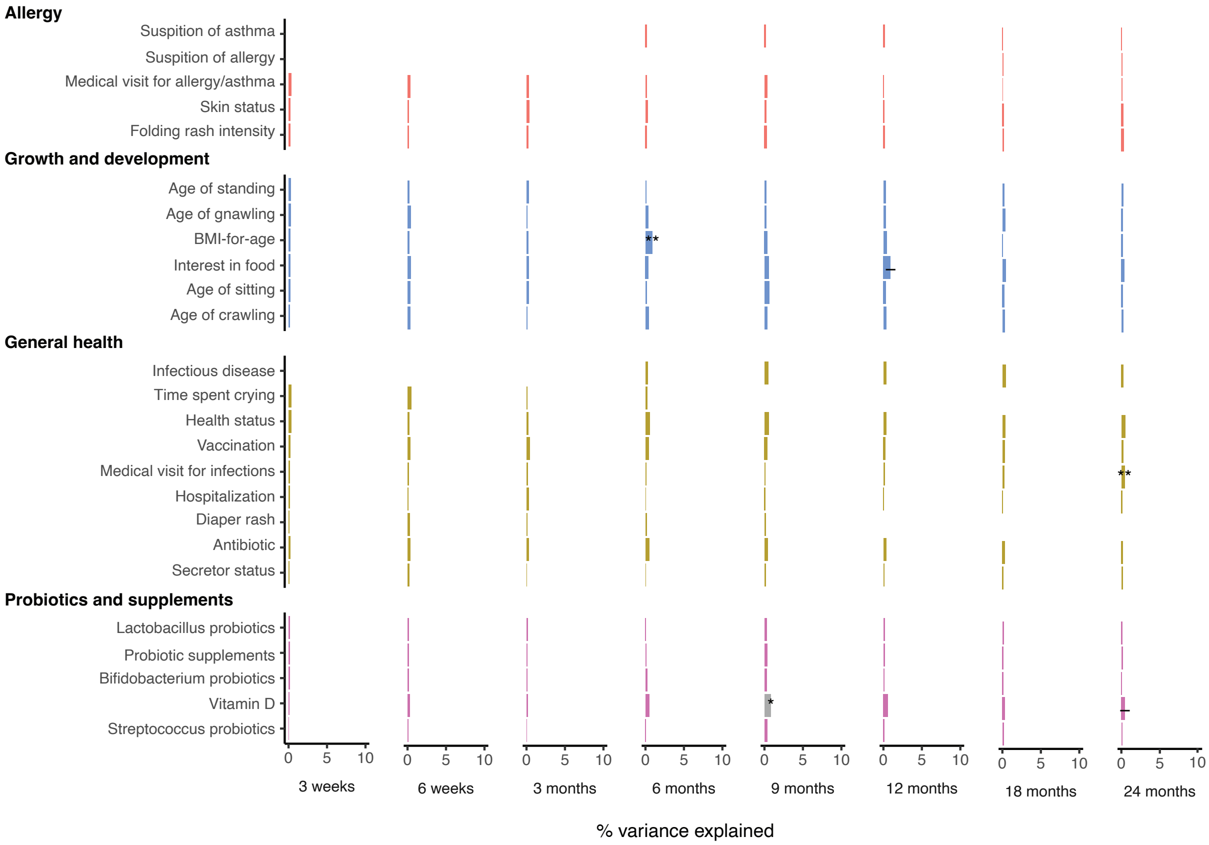


**Fig. S11 Variance explained by infant health and development by sampling age.** Results depict the coefficient of determination ($R^2$) from permutational multivariate analysis on Bray-Curtis distances after adjustment with extraction batch and number of reads. Asterisks mark false discovery rate corrected *P*-values. ***: *P*<0.001, **: *P*<0.01, *: *P*<0.05, -: P<0.1, all non-corrected *P*-values <0.05. Grey bars mark uneven beta-dispersion between the categories of a variable. A detailed description of the variables, their definitions and groupings are available in Supplemental File 1.

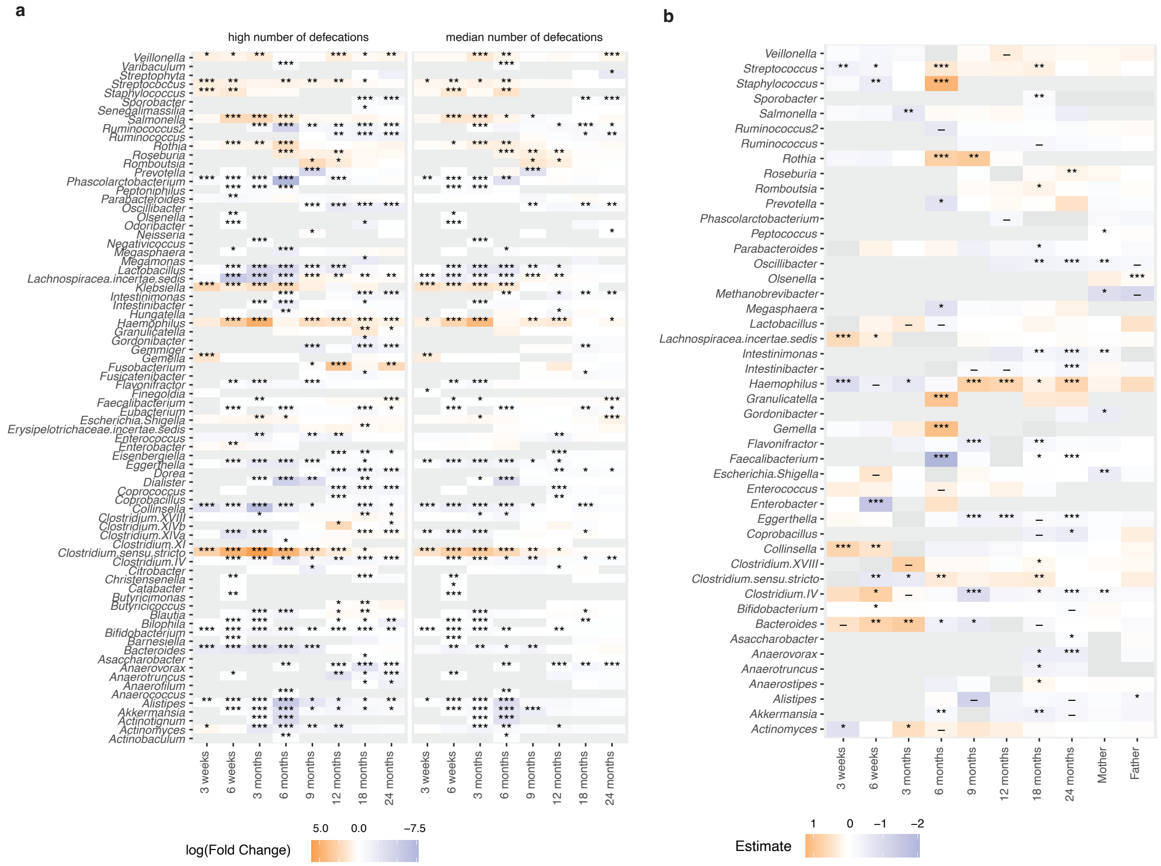


**Fig. S12 Associations between (a) defecation rate and (b stool consistency and relative abundances of different genera by sampling age.** For defecation rate, a three-class variable was created by sampling age and the lowest rate group was used as the reference. The stool consistency was treated as a continuous variable. The differential abundance tests were adjusted for the DNA extraction batch. Asterisks mark false discovery rate corrected *P*-values. ***: *P*<0.001, **: *P*<0.01, *: *P*<0.05, -: P<0.1, all non-corrected *P*-values <0.05. A detailed description of the variables, their definitions and groupings are available in Supplemental File 1.

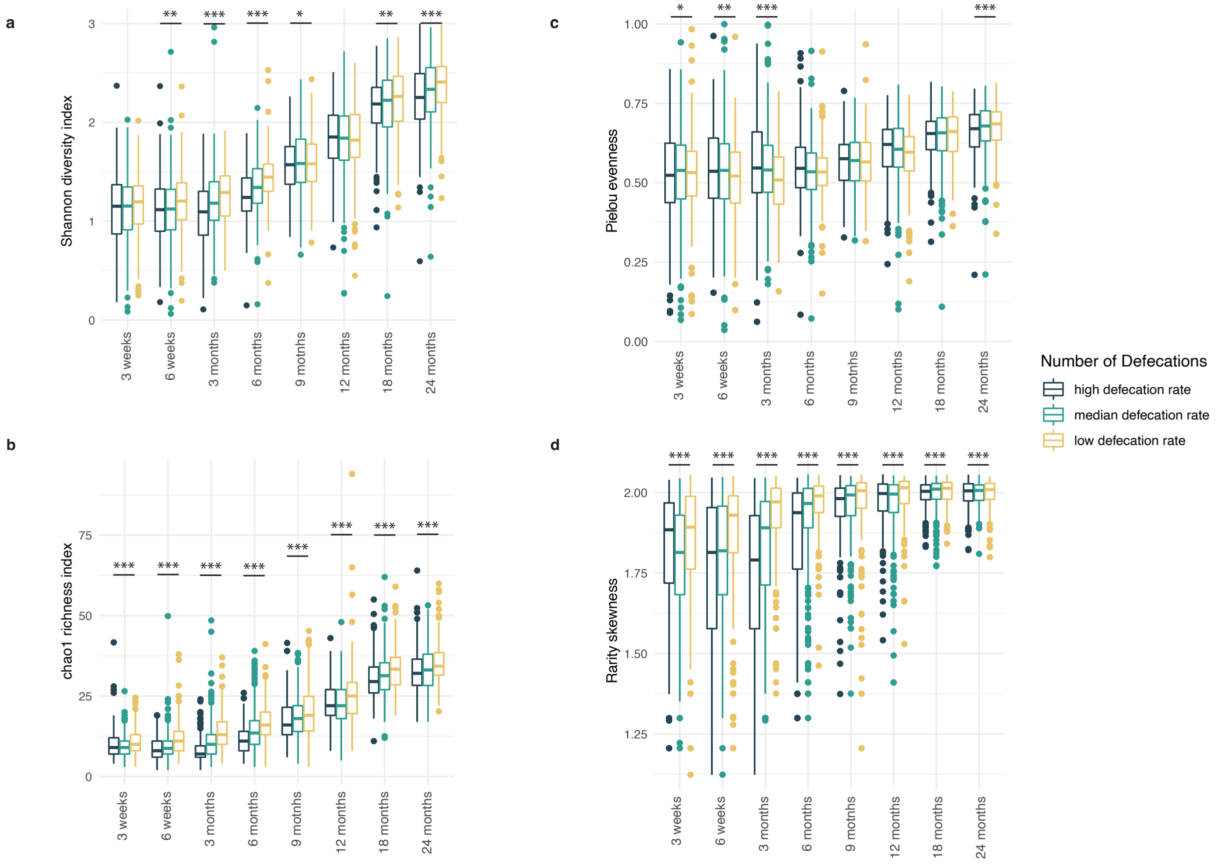


**Fig. S13 Associations between defecation rate and (a) alpha-diversity, (b) richness, (c) evenness, and (d) rarity by sampling age**. Asterisks mark *P*-values. ***: *P*<0.001, **: *P*<0.01, *: *P*<0.05, -: *P*<0.1, all non-corrected *P*-values <0.05. A detailed description of the variables, their definitions and groupings are available in Supplemental File 1.

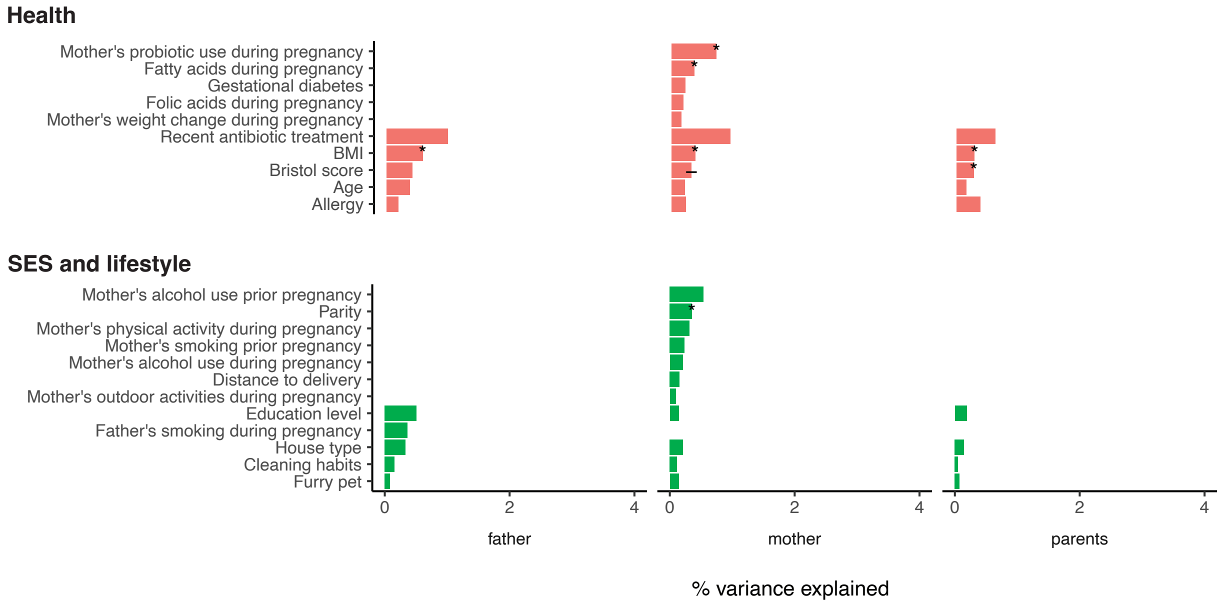


**Fig. S14 Variance explained in mothers' and fathers' samples and parental samples pooled together.** Results depict the coefficient of determination ($R^2$) from permutational multivariate analysis on Bray-Curtis distances after adjustment with extraction batch and number processed of reads. Asterisks mark false discovery rate corrected *P*-values. ***: *P*<0.001, **: *P*<0.01, *: *P*<0.05, -: *P*<0.1, all non-corrected P-values <0.05.

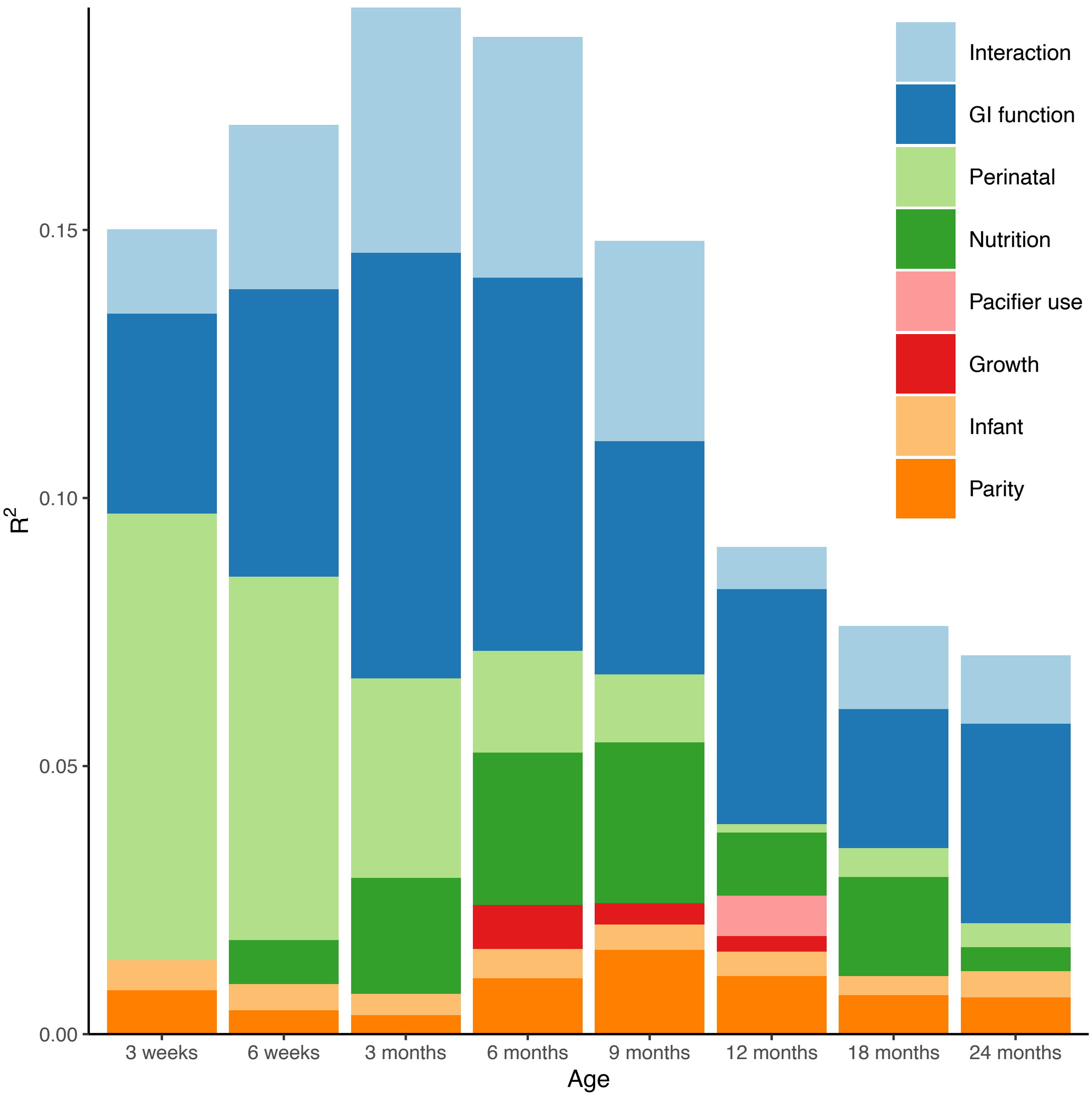


**Fig. S15 Total bacterial variation explained by biological variable groups by sampling age.** Variance explained derived from permutational multivariate models, confounded with DNA extraction batch, and number of processed reads, that were backward selected using Akaike information criteria. The variables were grouped based on their type and include relevant interactions. Only pacifier use, growth (WHO BMI z-score) and parity represent single variables. Interactions include the most important two-variable interactions (var1:var2) by sampling age and are detailed in Supplemental file 2. A detailed description of the variables, their definitions and groupings are available in Supplemental File 1. GI=gastrointestinal.

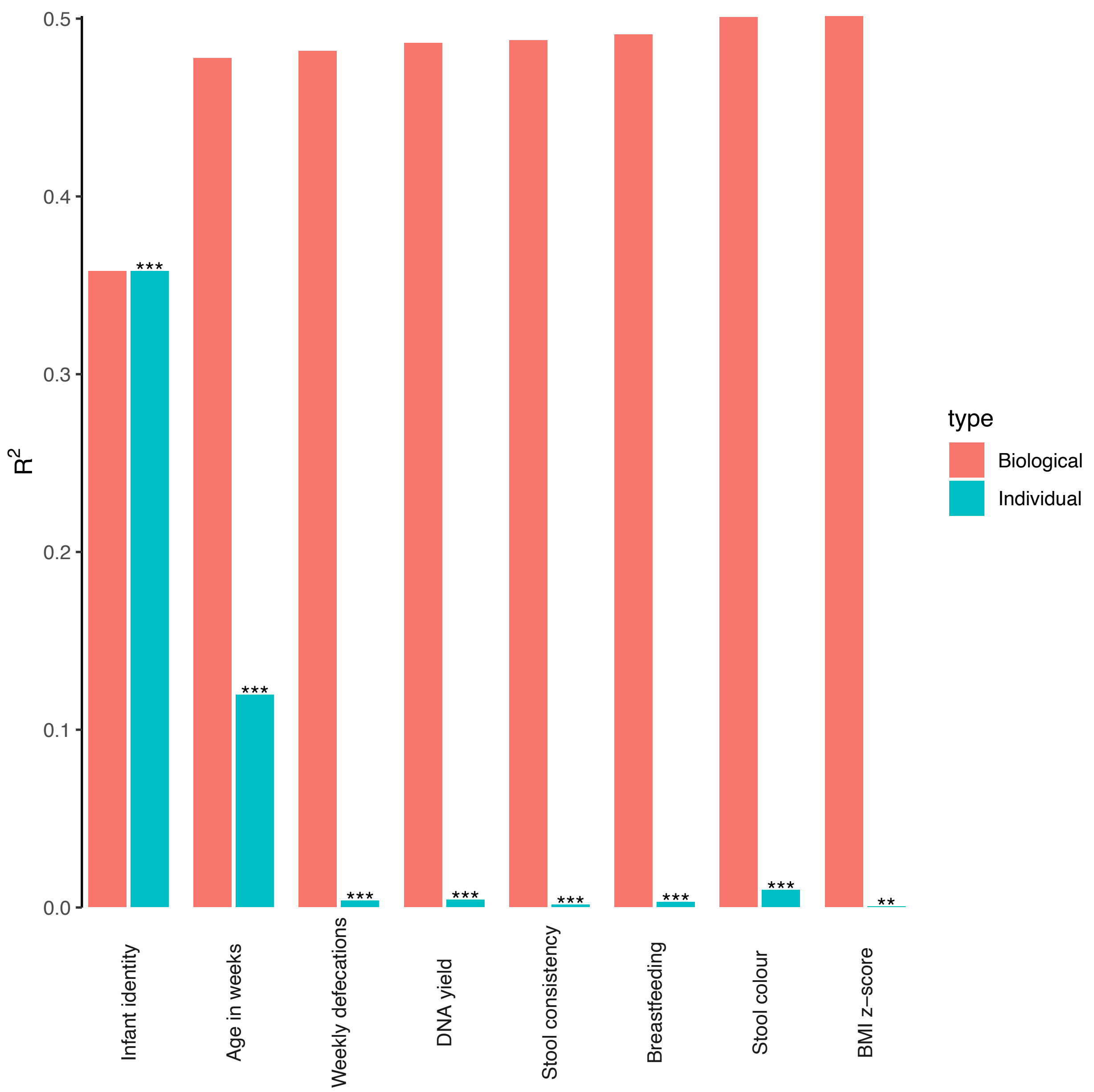


**Fig. S16 Total bacterial variation explained in all infant samples from the first 2 years of life.** Variance explained derived from permutational multivariate models, confounded with the number of processed reads, that were backward selected using Akaike information criteria. Red bars represent the total cumulative effect of the biological variables, and cyan bars mark the contribution of individual variables to the overall model. Asterisks mark *P*-values in the cumulative models. ***: $P<0.001$, **: $P<0.01$, *: $P<0.05$. A detailed description of the variables, their definitions and groupings are available in Supplemental File 1.

# References


1. Jokela R, Korpela K, Jian C, Dikareva E, Nikkonen A, Saisto T, et al. Quantitative insights into effects of intrapartum antibiotics and birth mode on infant gut microbiota in relation to well-being during the first year of life. Gut Microbes. 2022 Dec 31;14(1):2095775.

2. Vandeputte D, Falony G, Vieira-Silva S, Tito RY, Joossens M, Raes J. Stool consistency is strongly associated with gut microbiota richness and composition, enterotypes and bacterial growth rates. Gut. 2016 Jan 1;65(1):57.

3. Milani C, Duranti S, Bottacini F, Casey E, Turroni F, Mahony J, et al. The First Microbial Colonizers of the Human Gut: Composition, Activities, and Health Implications of the Infant Gut Microbiota. Microbiol Mol Biol Rev. 2017 Nov 8;81(4):e00036-17.

4. Reynoso-García J, Miranda-Santiago AE, Meléndez-Vázquez NM, Acosta-Pagán K, Sánchez-Rosado M, Díaz-Rivera J, et al. A complete guide to human microbiomes: Body niches, transmission, development, dysbiosis, and restoration. Frontiers in Systems Biology [Internet]. 2022 [cited 2023 Jan 11];2. Available from: https://www.frontiersin.org/articles/10.3389/fsysb.2022.951403

5. Eisenhofer R, Minich JJ, Marotz C, Cooper A, Knight R, Weyrich LS. Contamination in Low Microbial Biomass Microbiome Studies: Issues and Recommendations. Trends in Microbiology. 2019 Feb 1;27(2):105–17.
